# Supplementary figures and images for: Dental pulp stem cells ameliorate D-galactose-induced cardiac ageing in rats
Source: PeerJ. 2024 May 21;12:e17299. doi: 10.7717/peerj.17299 (PMC11127642; doi:10.7717/peerj.17299)

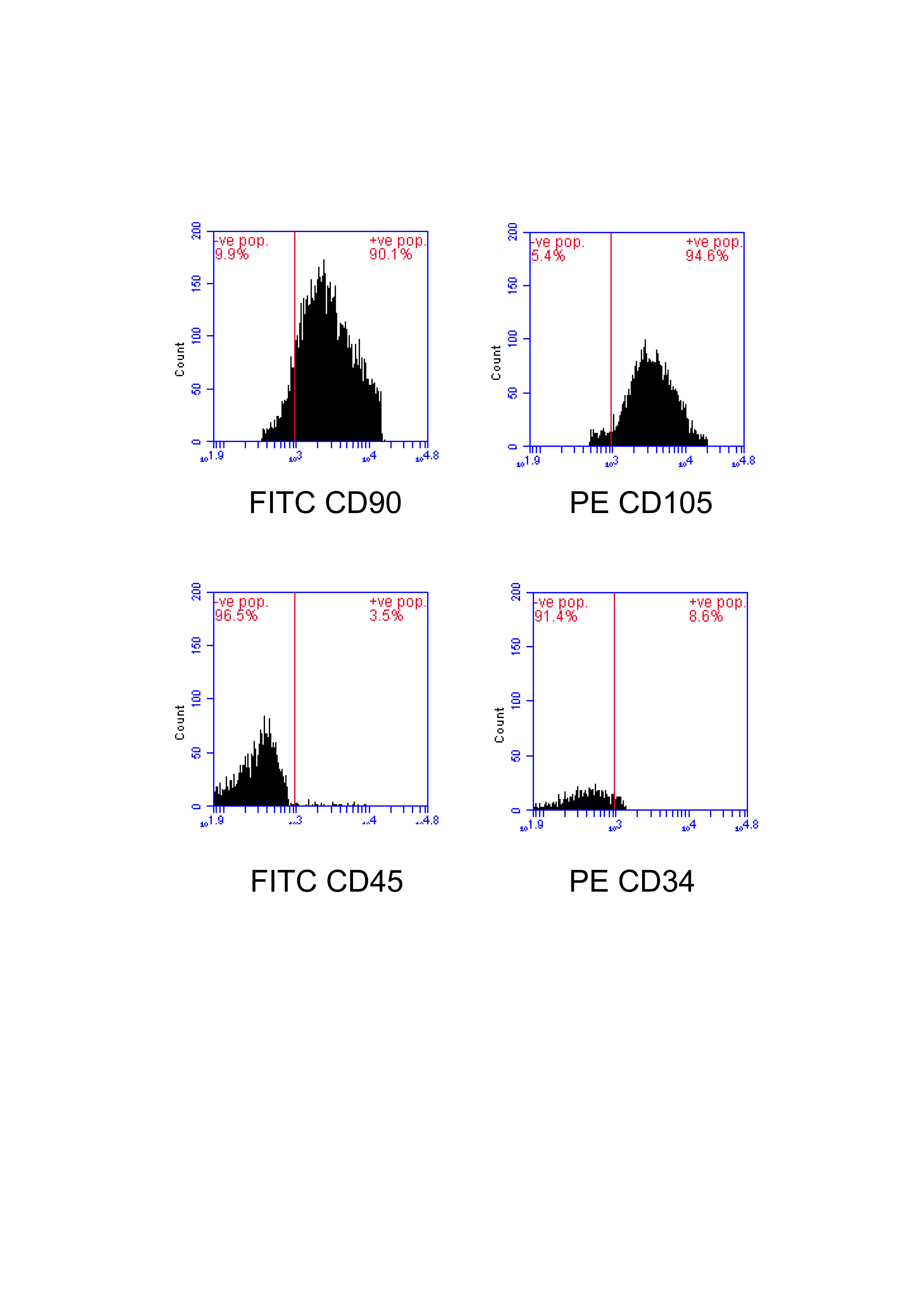

Supplement: Figure S1 [file peerj-12-17299-s001.png]

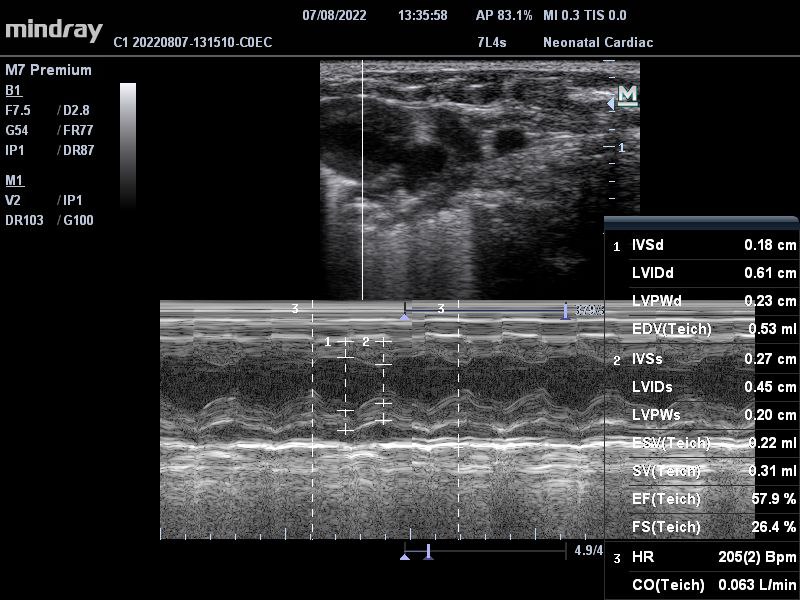

Supplement: Supplemental Information 4 [file peerj-12-17299-s004.zip › Raw data_Cardiac function/Control group/Control 2.jpg]

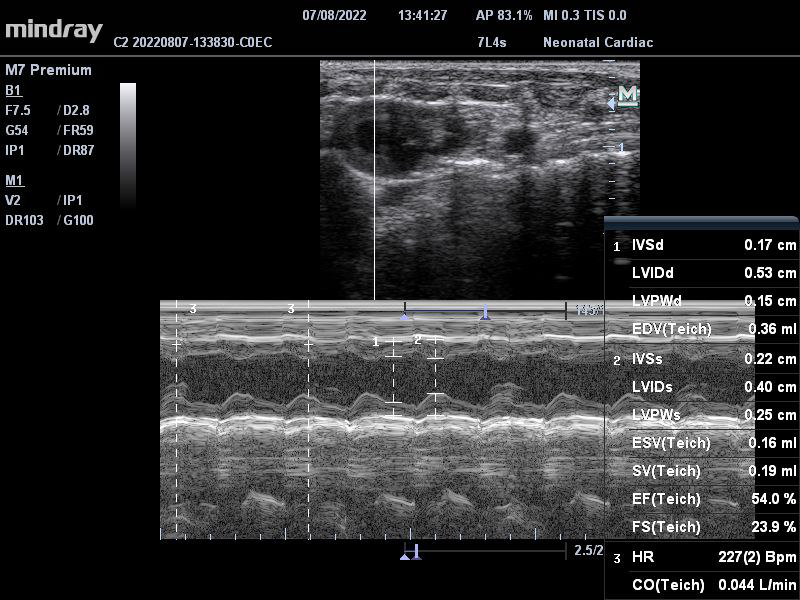

Supplement: Supplemental Information 4 [file peerj-12-17299-s004.zip › Raw data_Cardiac function/Control group/Control 1.png]

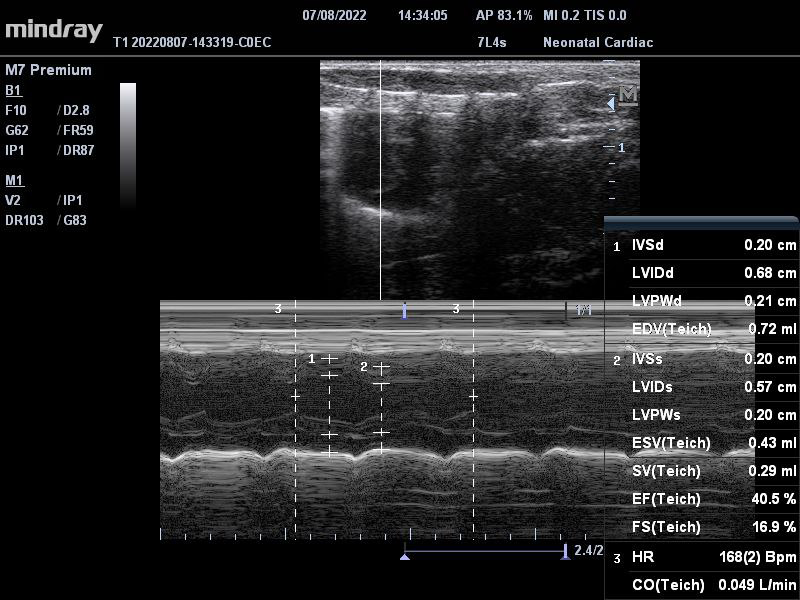

Supplement: Supplemental Information 4 [file peerj-12-17299-s004.zip › Raw data_Cardiac function/D-galactose+DPSCs/D-galactose+DPSCs 1.png]

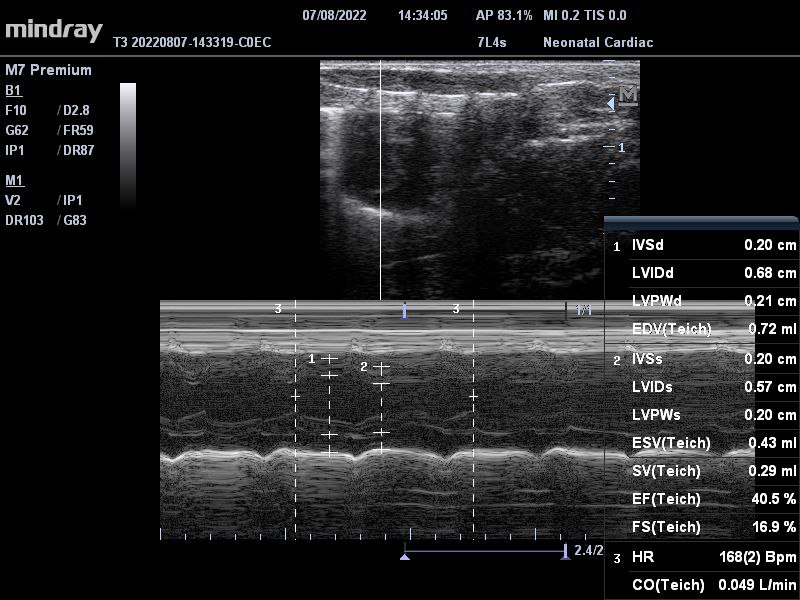

Supplement: Supplemental Information 4 [file peerj-12-17299-s004.zip › Raw data_Cardiac function/D-galactose+DPSCs/D-galactose+DPSCs 3.jpg]

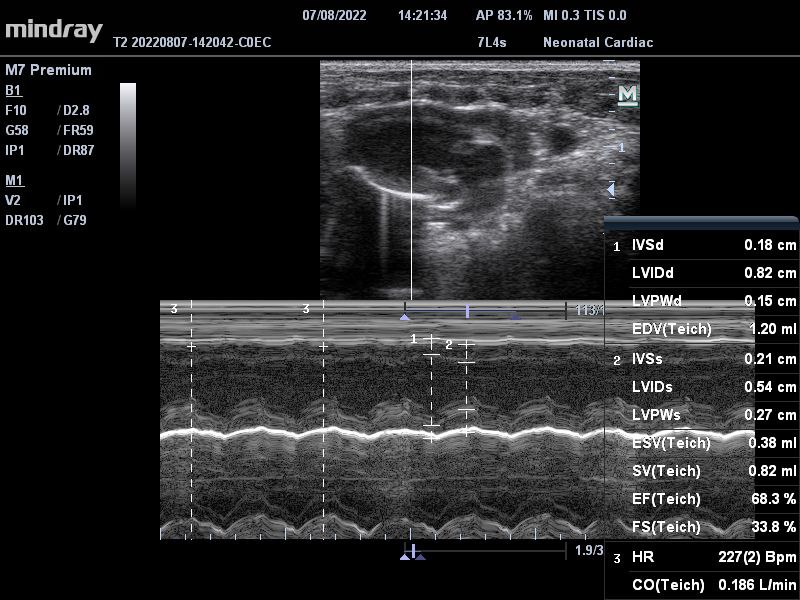

Supplement: Supplemental Information 4 [file peerj-12-17299-s004.zip › Raw data_Cardiac function/D-galactose+DPSCs/D-galactose+DPSCs 2.jpg]

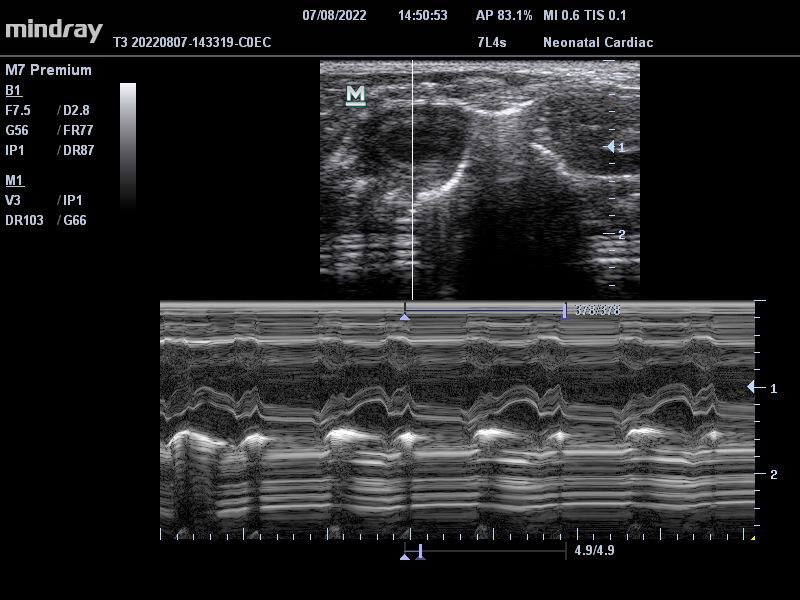

Supplement: Supplemental Information 4 [file peerj-12-17299-s004.zip › Raw data_Cardiac function/D-galactose+DPSCs/D-galactose+DPSCs 5.jpg]

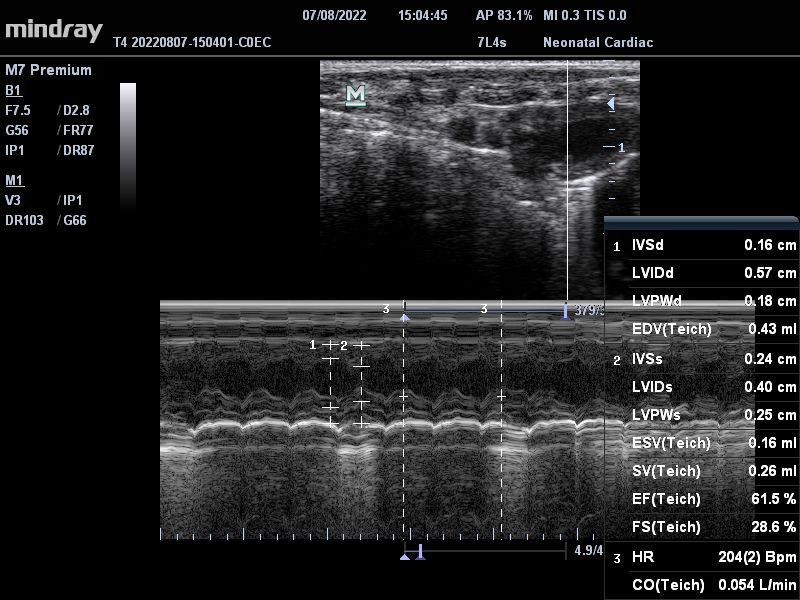

Supplement: Supplemental Information 4 [file peerj-12-17299-s004.zip › Raw data_Cardiac function/D-galactose+DPSCs/D-galactose+DPSCs 4.jpg]

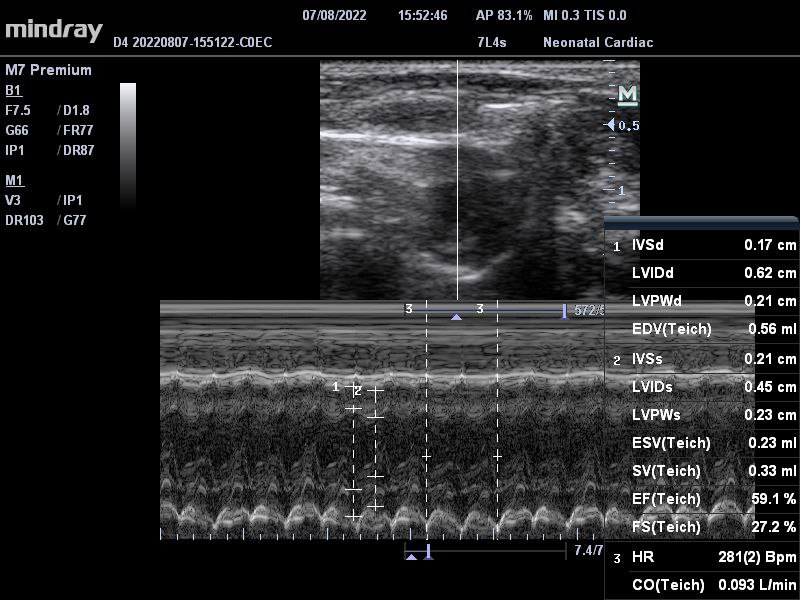

Supplement: Supplemental Information 4 [file peerj-12-17299-s004.zip › Raw data_Cardiac function/D-galactose group/D-galactose 4.jpg]

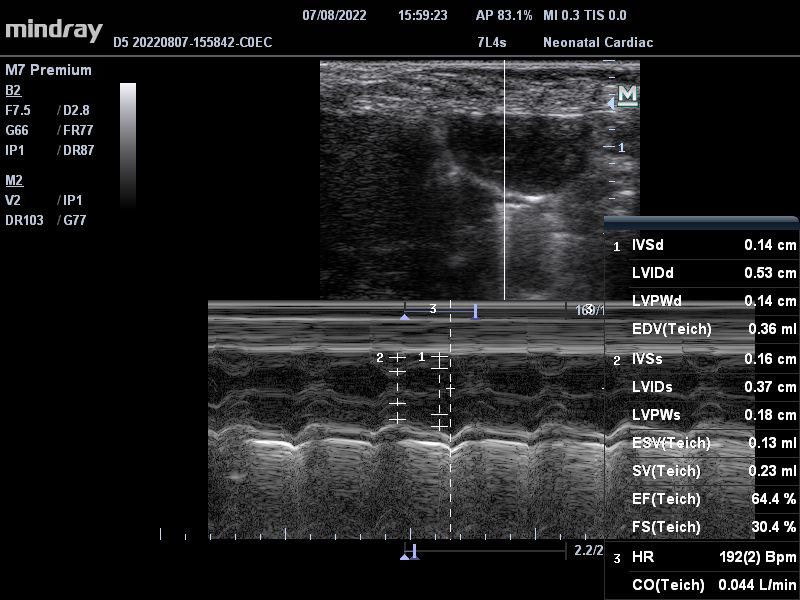

Supplement: Supplemental Information 4 [file peerj-12-17299-s004.zip › Raw data_Cardiac function/D-galactose group/D-galactose 5.jpg]

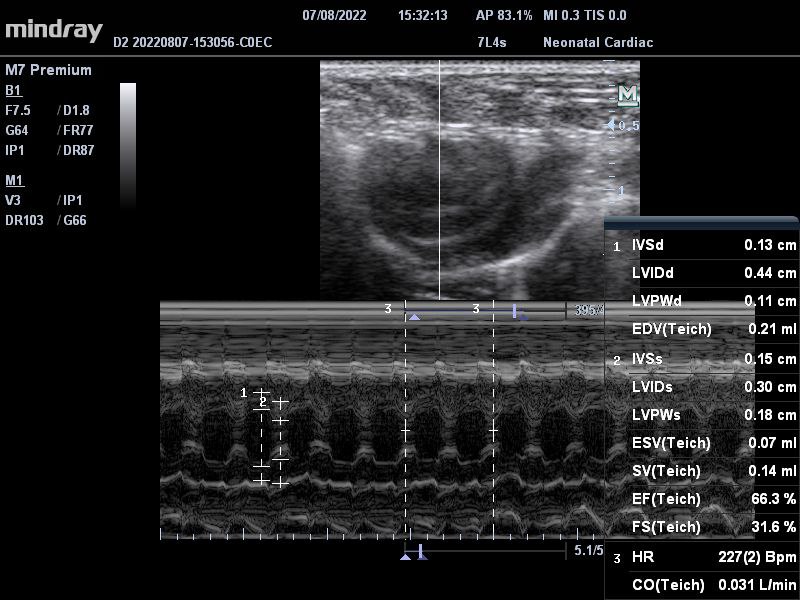

Supplement: Supplemental Information 4 [file peerj-12-17299-s004.zip › Raw data_Cardiac function/D-galactose group/D-galactose 2.jpg]

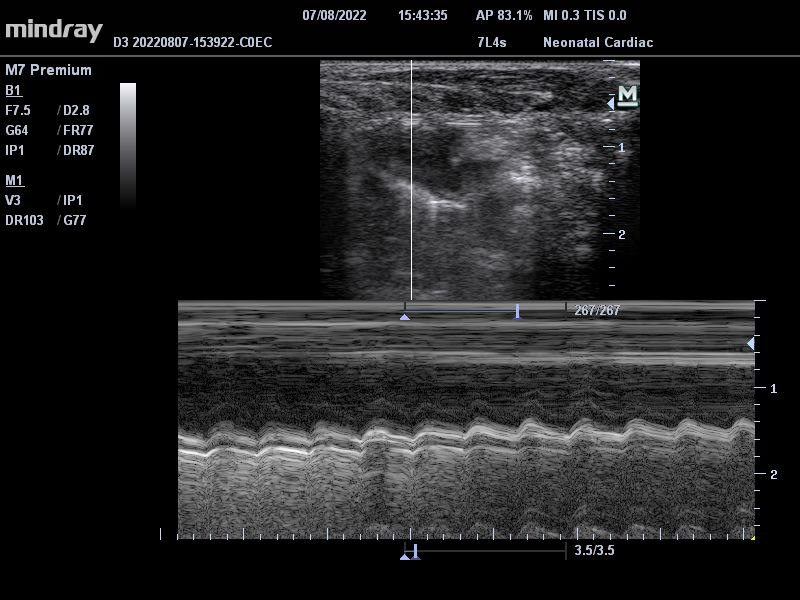

Supplement: Supplemental Information 4 [file peerj-12-17299-s004.zip › Raw data_Cardiac function/D-galactose group/D-galactose 3.jpg]

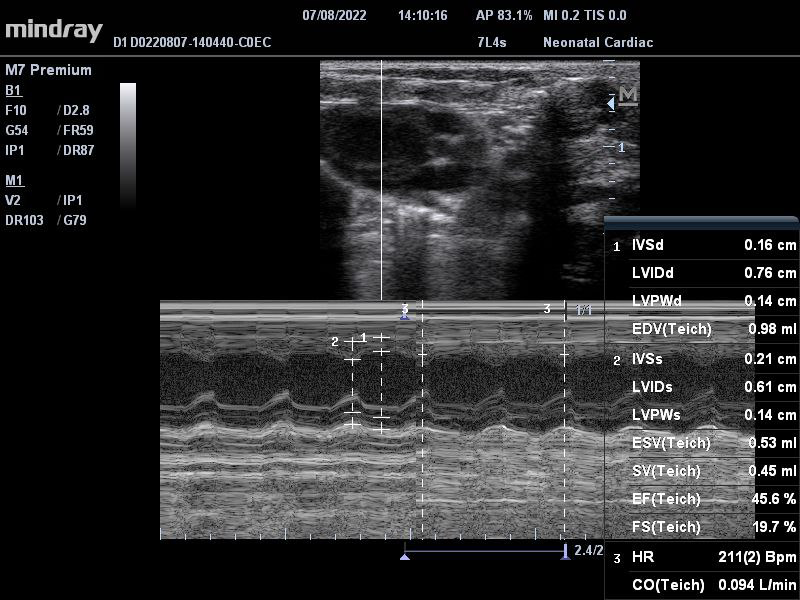

Supplement: Supplemental Information 4 [file peerj-12-17299-s004.zip › Raw data_Cardiac function/D-galactose group/D-galactose 1.png]

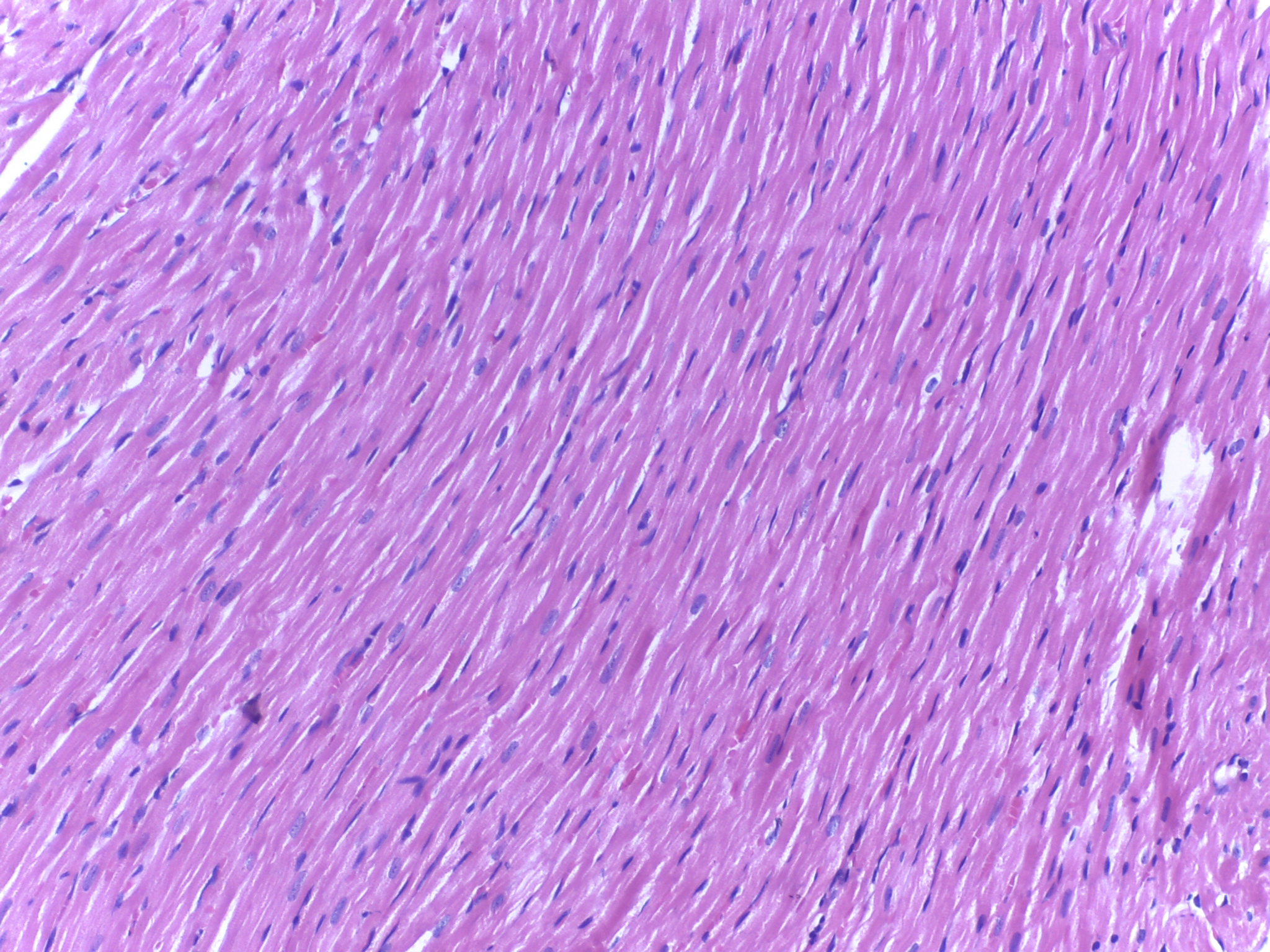

Supplement: Supplemental Information 5 [file peerj-12-17299-s005.zip › Raw Data_Histology/Control group/Control-4.jpg]

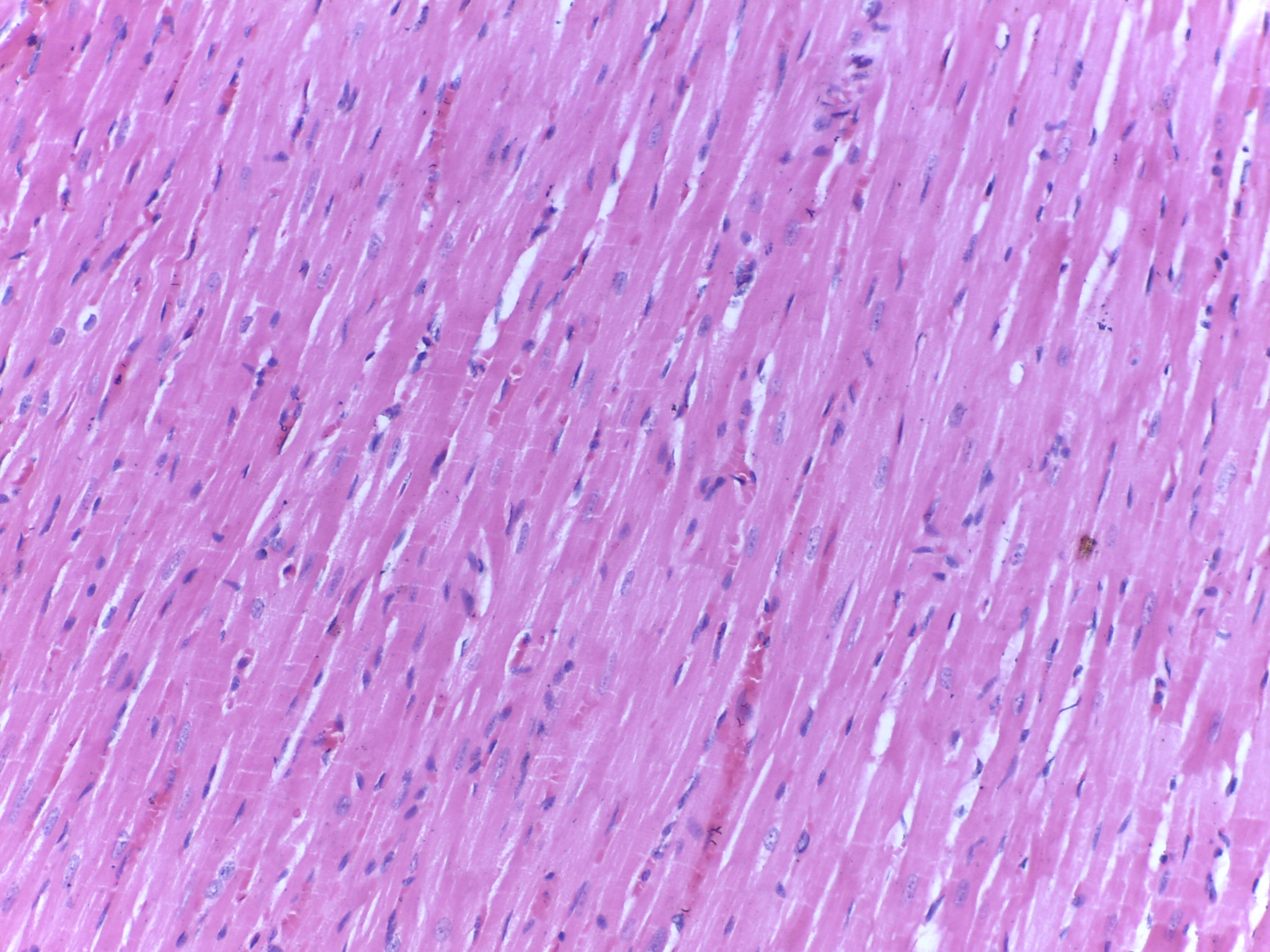

Supplement: Supplemental Information 5 [file peerj-12-17299-s005.zip › Raw Data_Histology/Control group/Control-3.jpg]

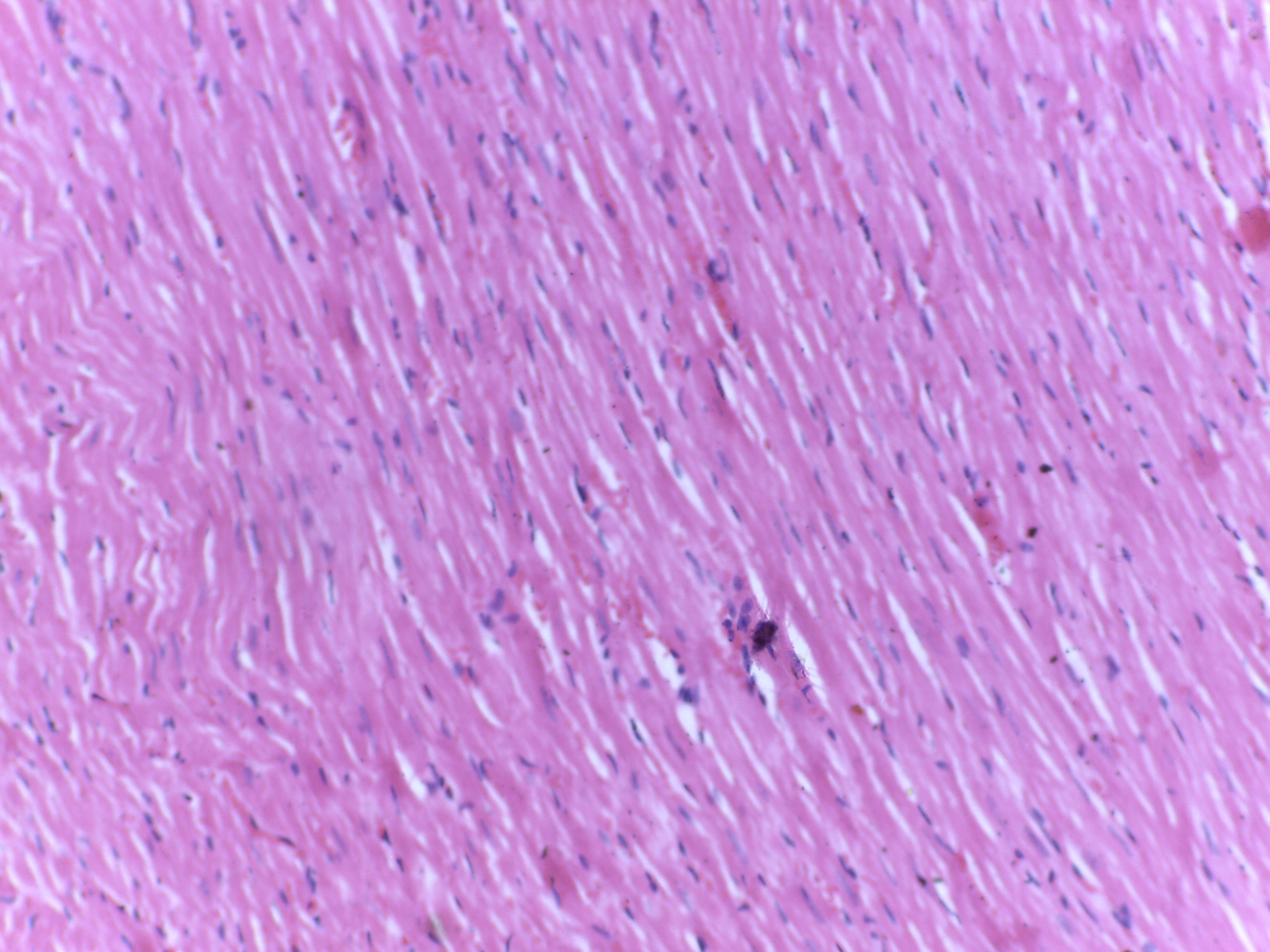

Supplement: Supplemental Information 5 [file peerj-12-17299-s005.zip › Raw Data_Histology/Control group/Control-2.jpg]

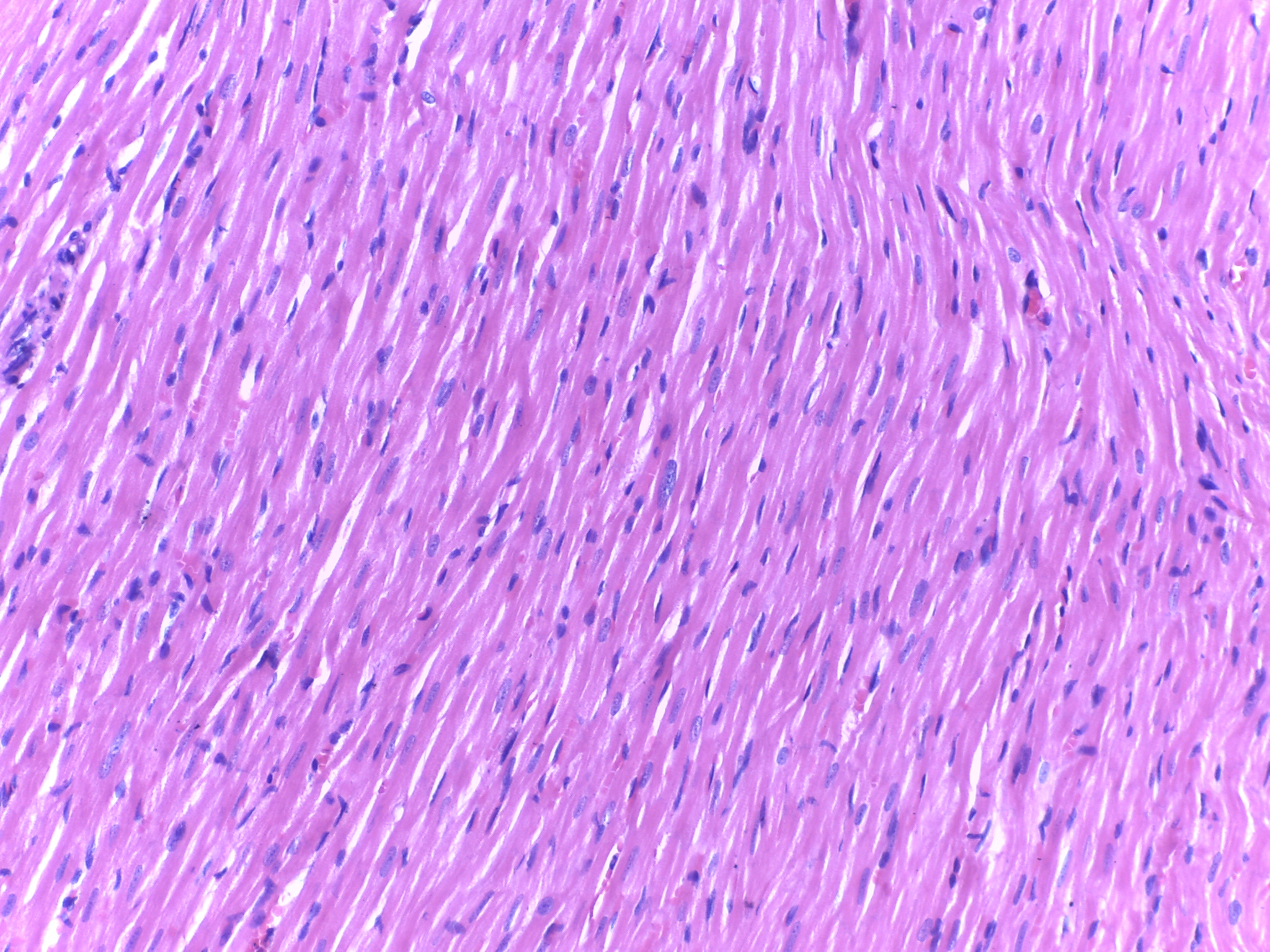

Supplement: Supplemental Information 5 [file peerj-12-17299-s005.zip › Raw Data_Histology/Control group/control-1.jpg]

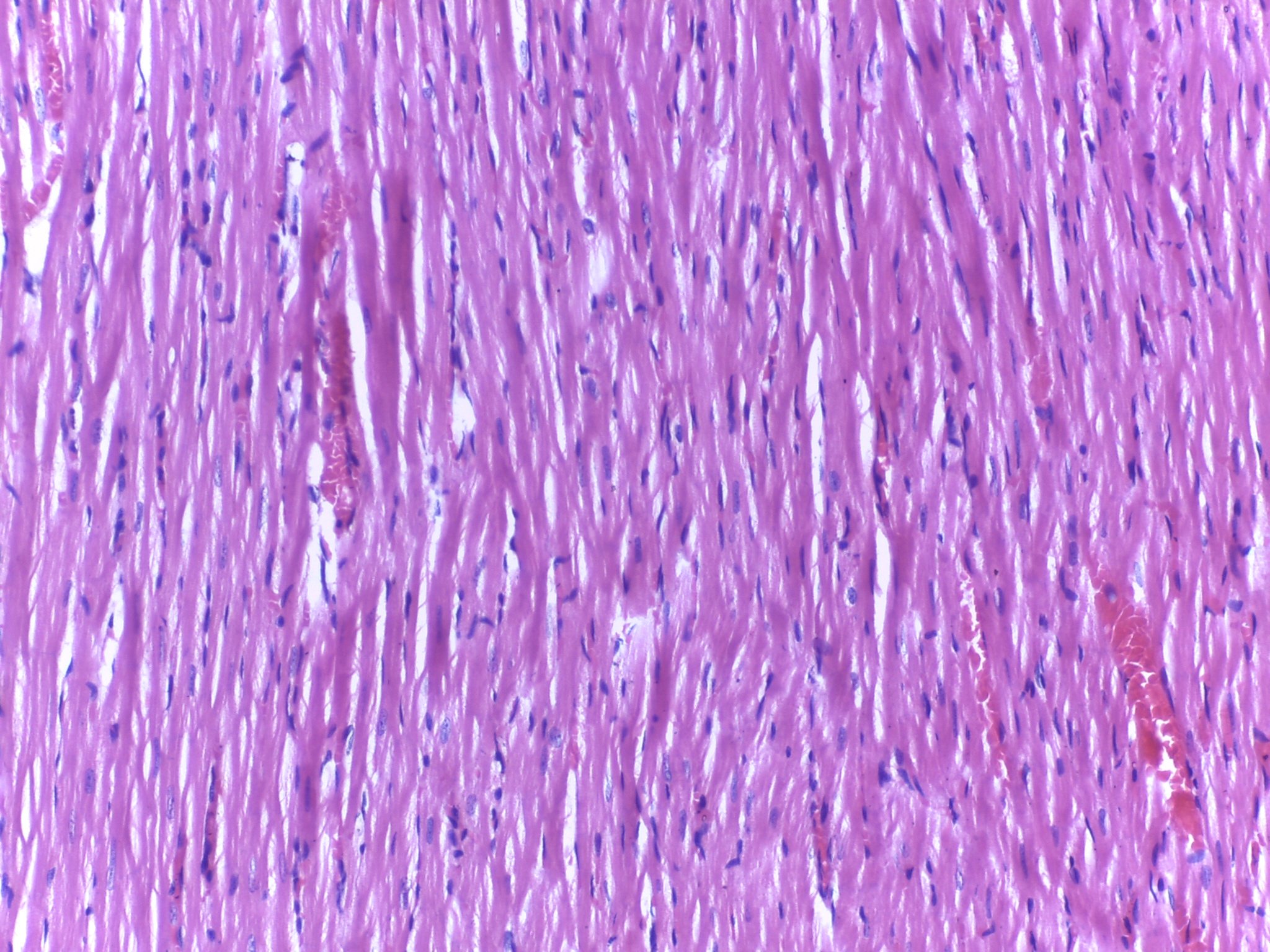

Supplement: Supplemental Information 5 [file peerj-12-17299-s005.zip › Raw Data_Histology/D galactose+DPSCs group/D-gal-MSC-2.jpg]

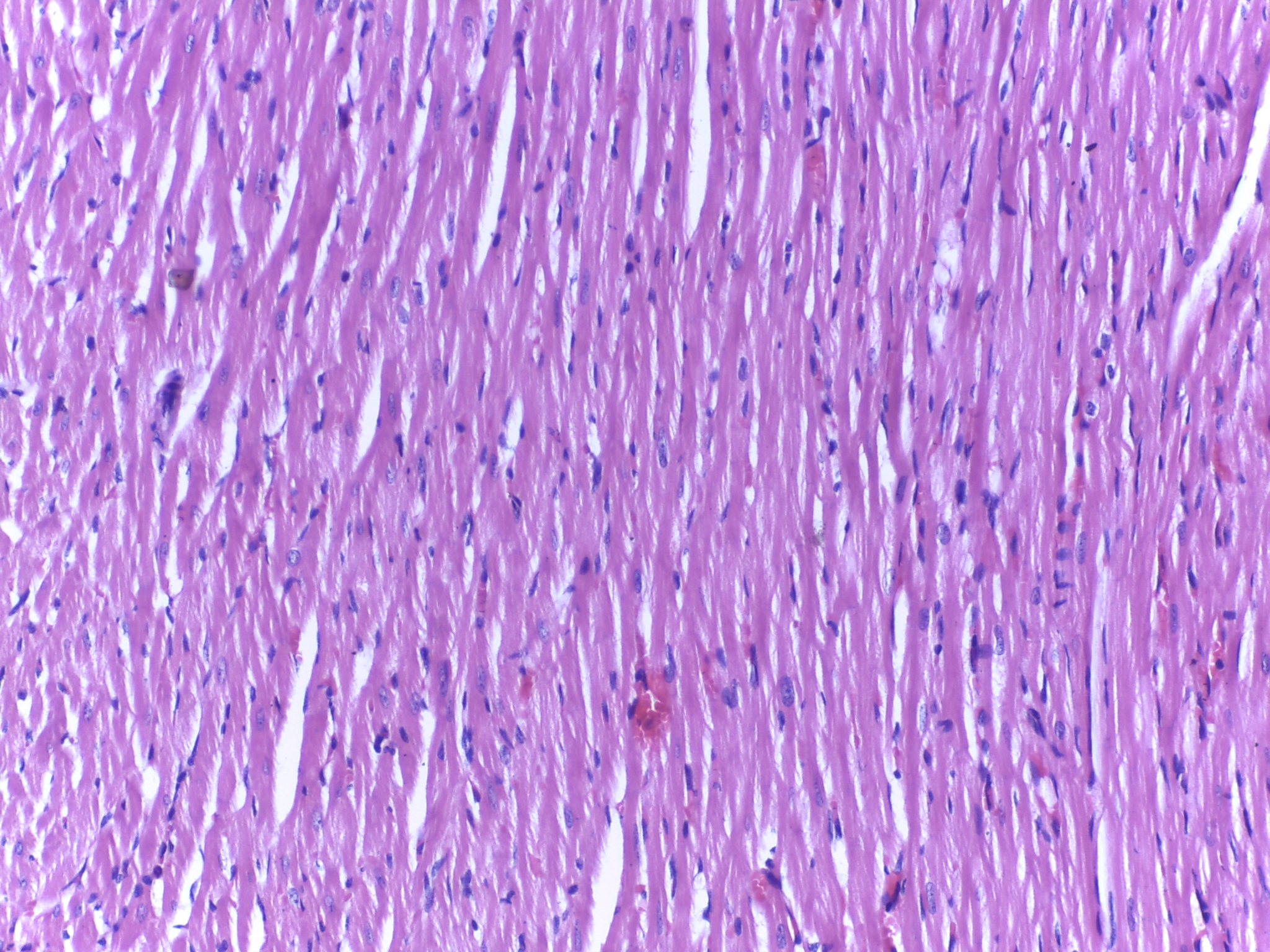

Supplement: Supplemental Information 5 [file peerj-12-17299-s005.zip › Raw Data_Histology/D galactose+DPSCs group/D-gal-MSC-1.jpg]

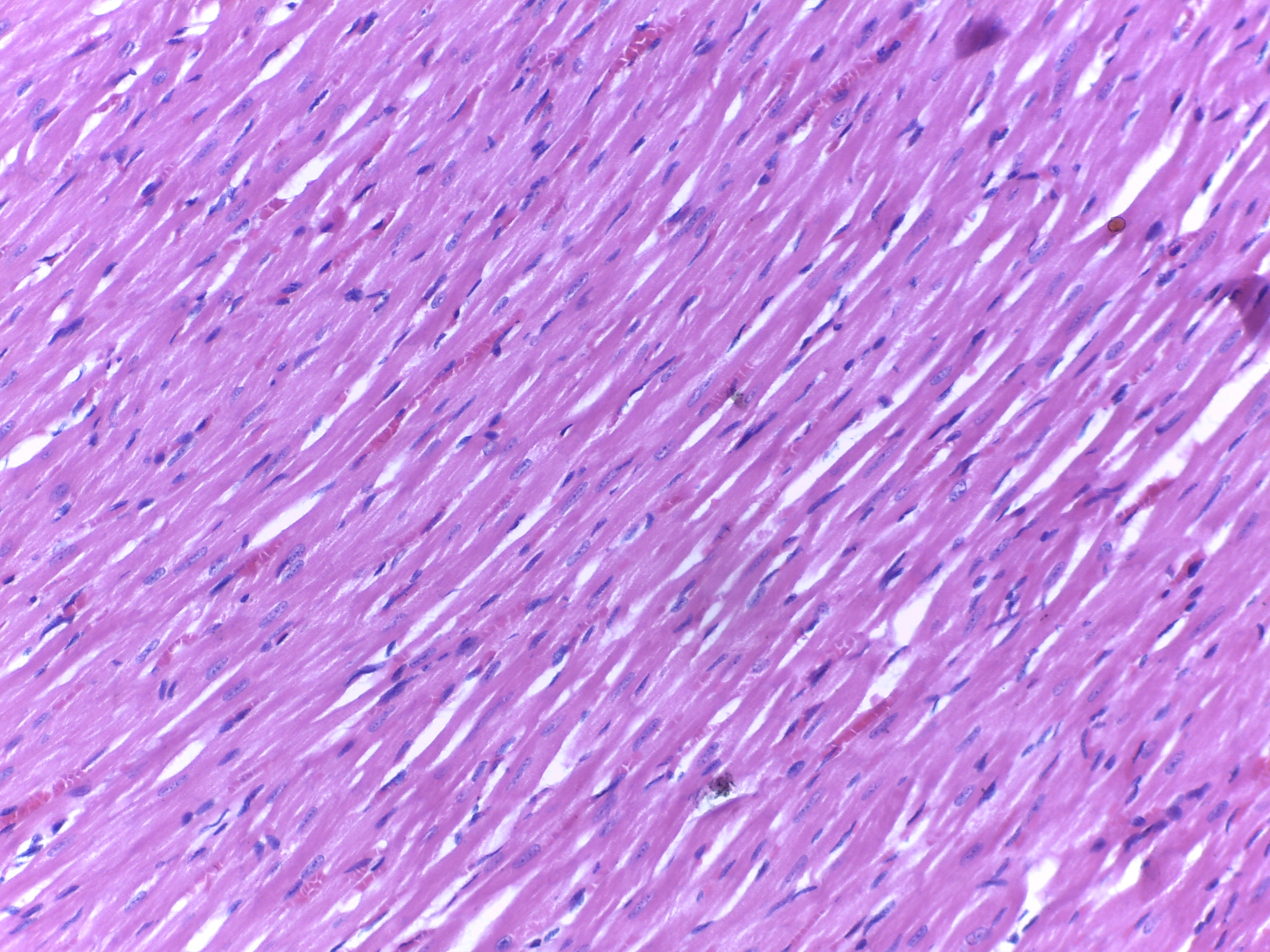

Supplement: Supplemental Information 5 [file peerj-12-17299-s005.zip › Raw Data_Histology/D galactose+DPSCs group/D-gal-MSCs-4.jpg]

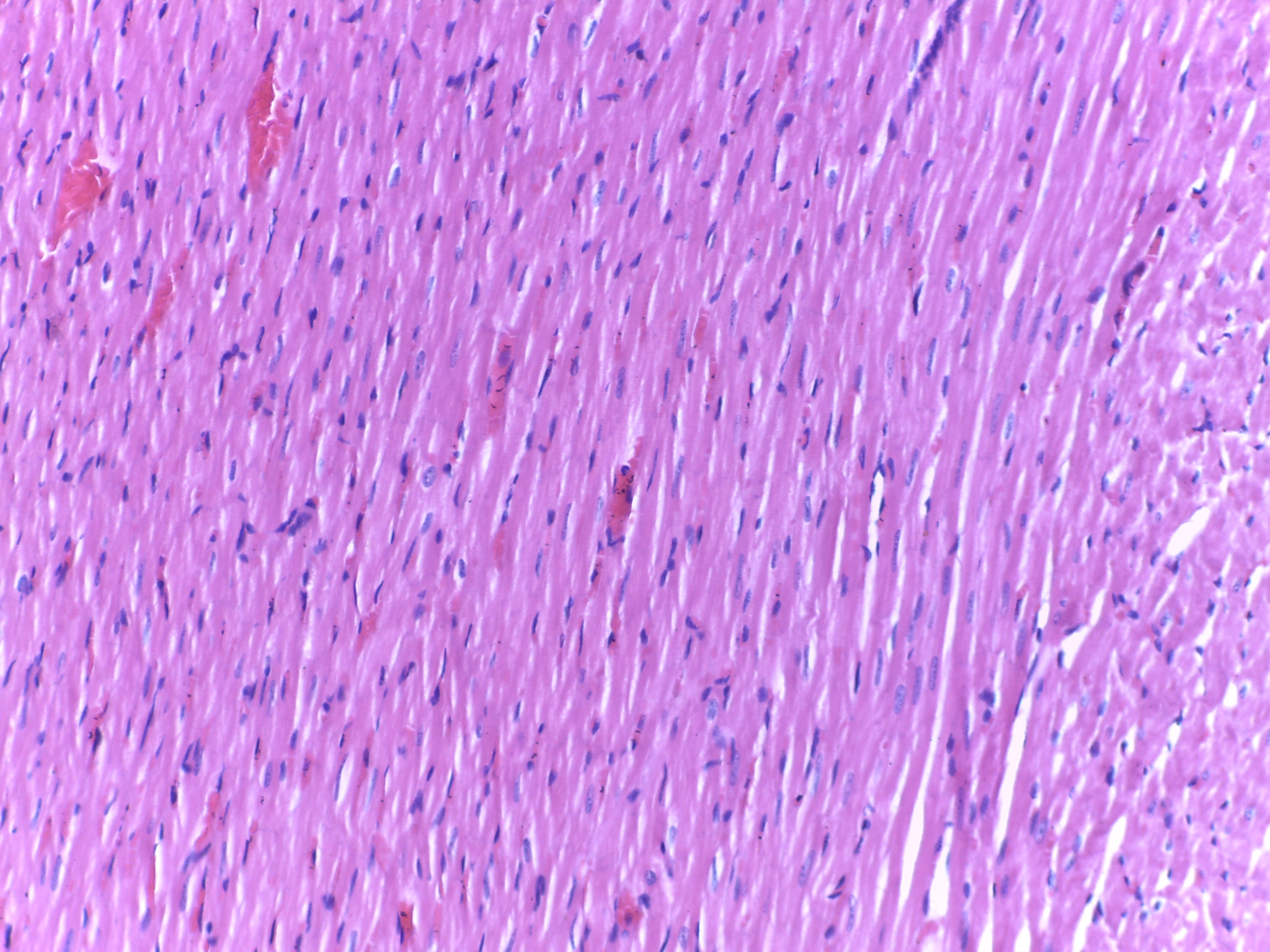

Supplement: Supplemental Information 5 [file peerj-12-17299-s005.zip › Raw Data_Histology/D galactose+DPSCs group/D-gal-MSCs-3.jpg]

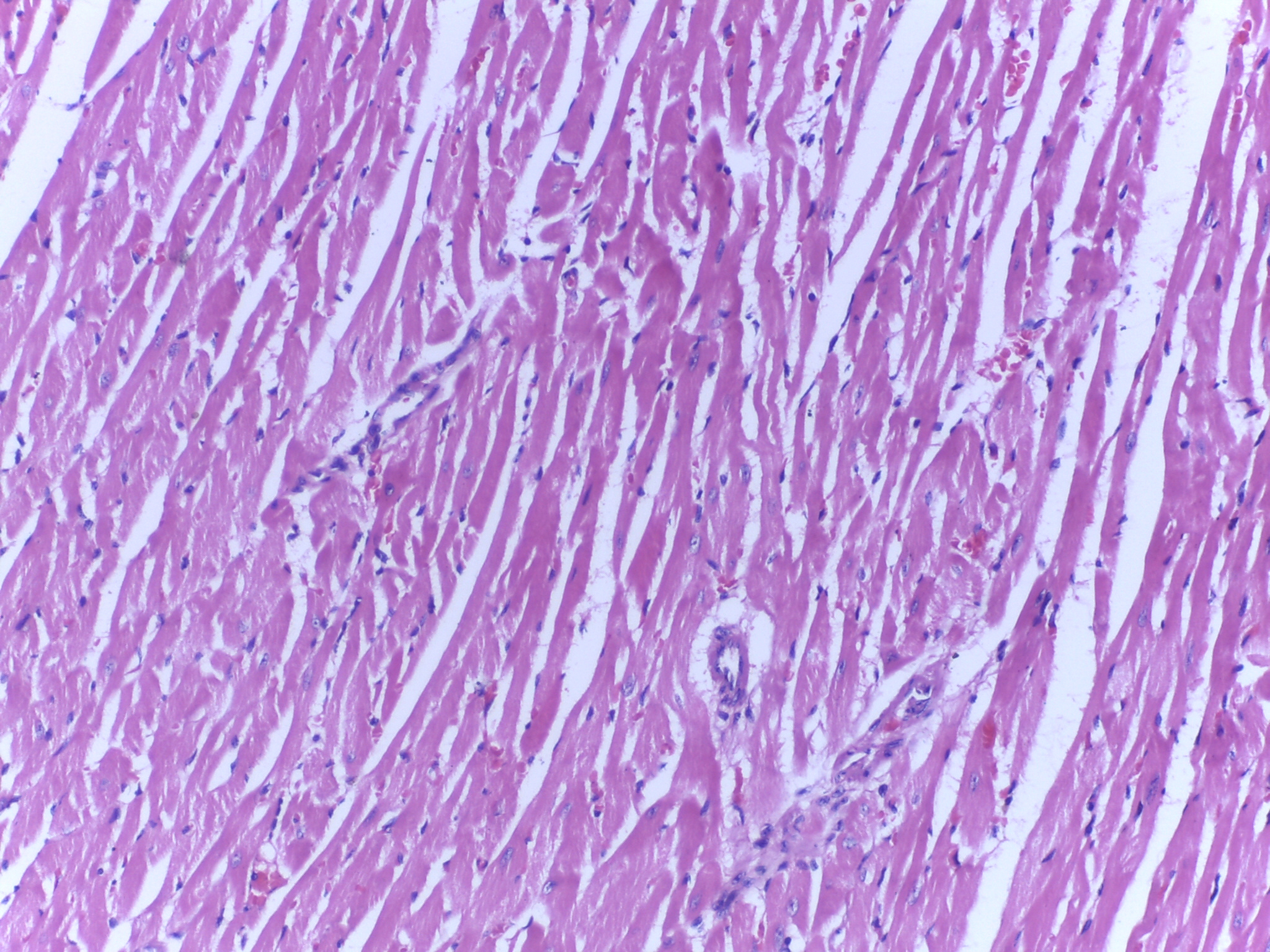

Supplement: Supplemental Information 5 [file peerj-12-17299-s005.zip › Raw Data_Histology/D galactose group/D-gal-3.jpg]

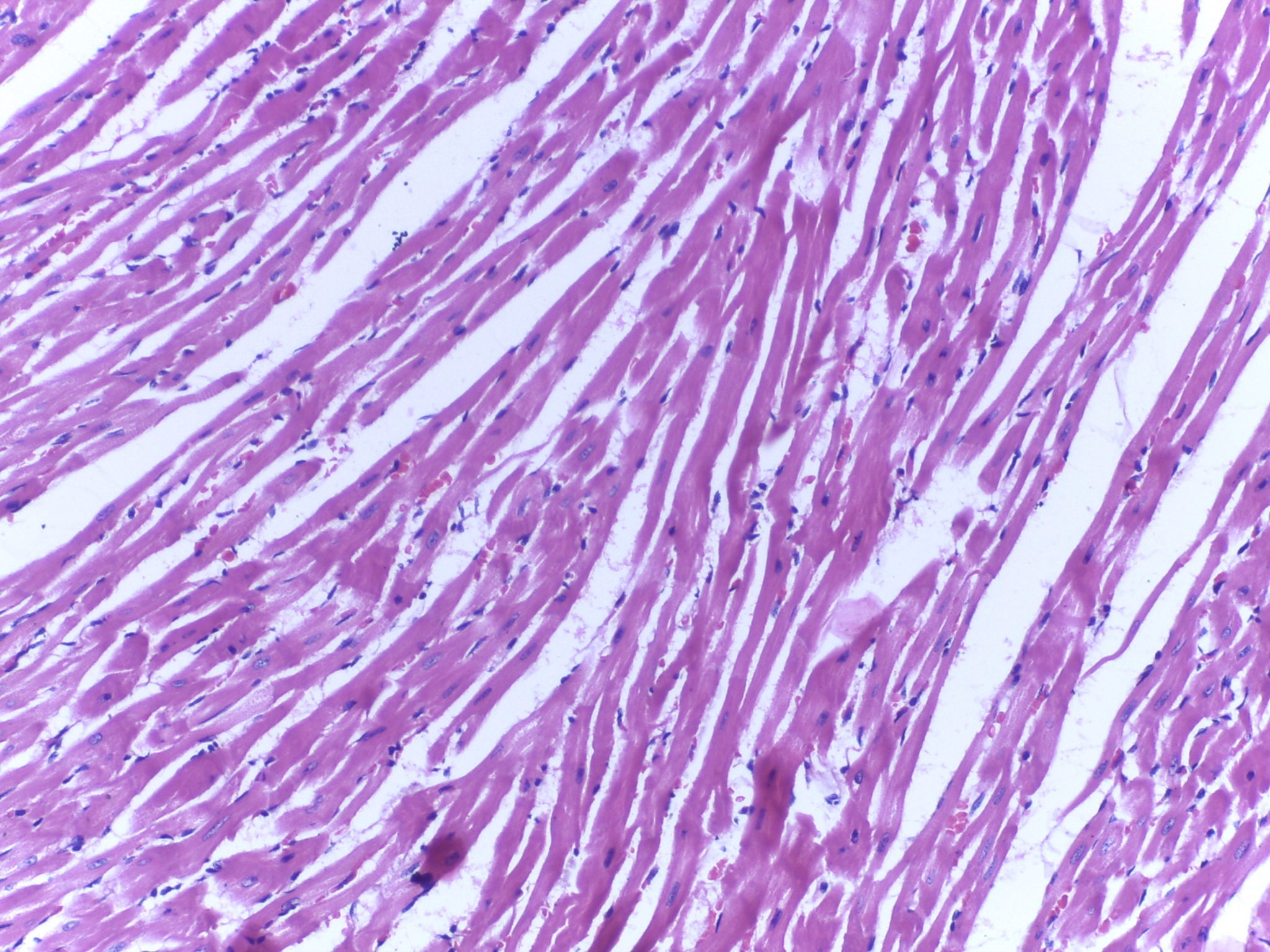

Supplement: Supplemental Information 5 [file peerj-12-17299-s005.zip › Raw Data_Histology/D galactose group/D-gal-2.jpg]

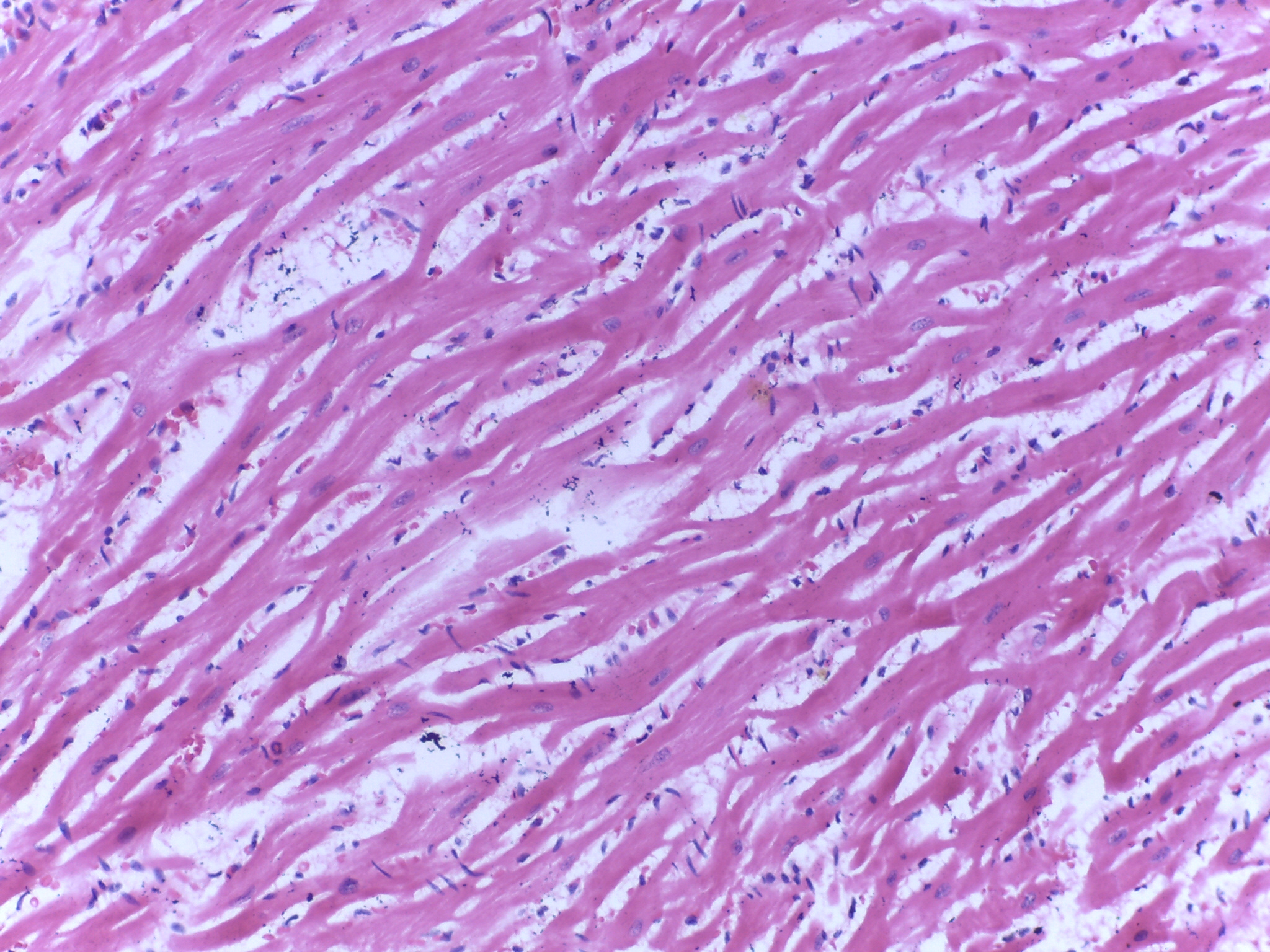

Supplement: Supplemental Information 5 [file peerj-12-17299-s005.zip › Raw Data_Histology/D galactose group/D-gal-1.jpg]

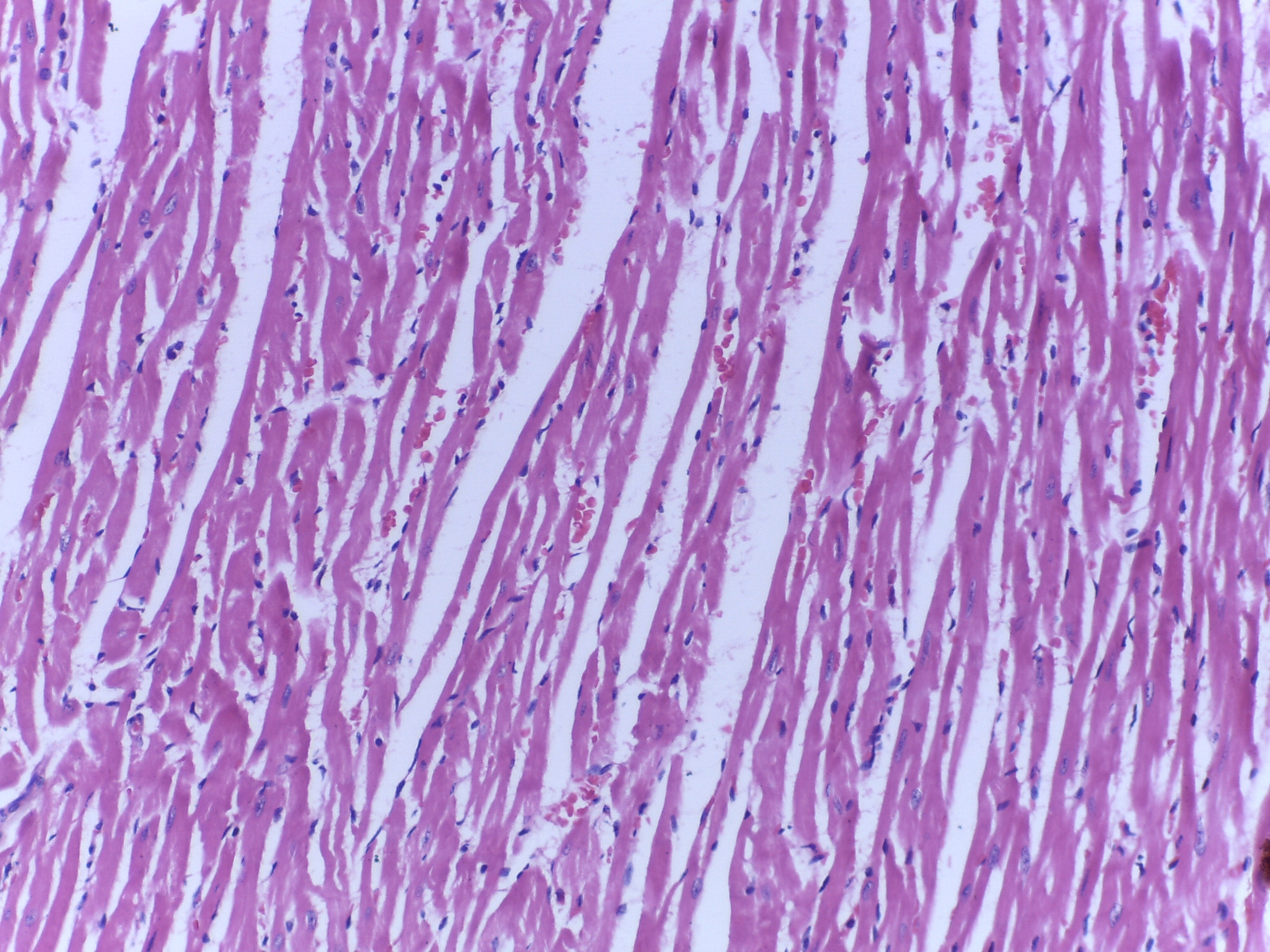

Supplement: Supplemental Information 5 [file peerj-12-17299-s005.zip › Raw Data_Histology/D galactose group/D-gal-4.jpg]

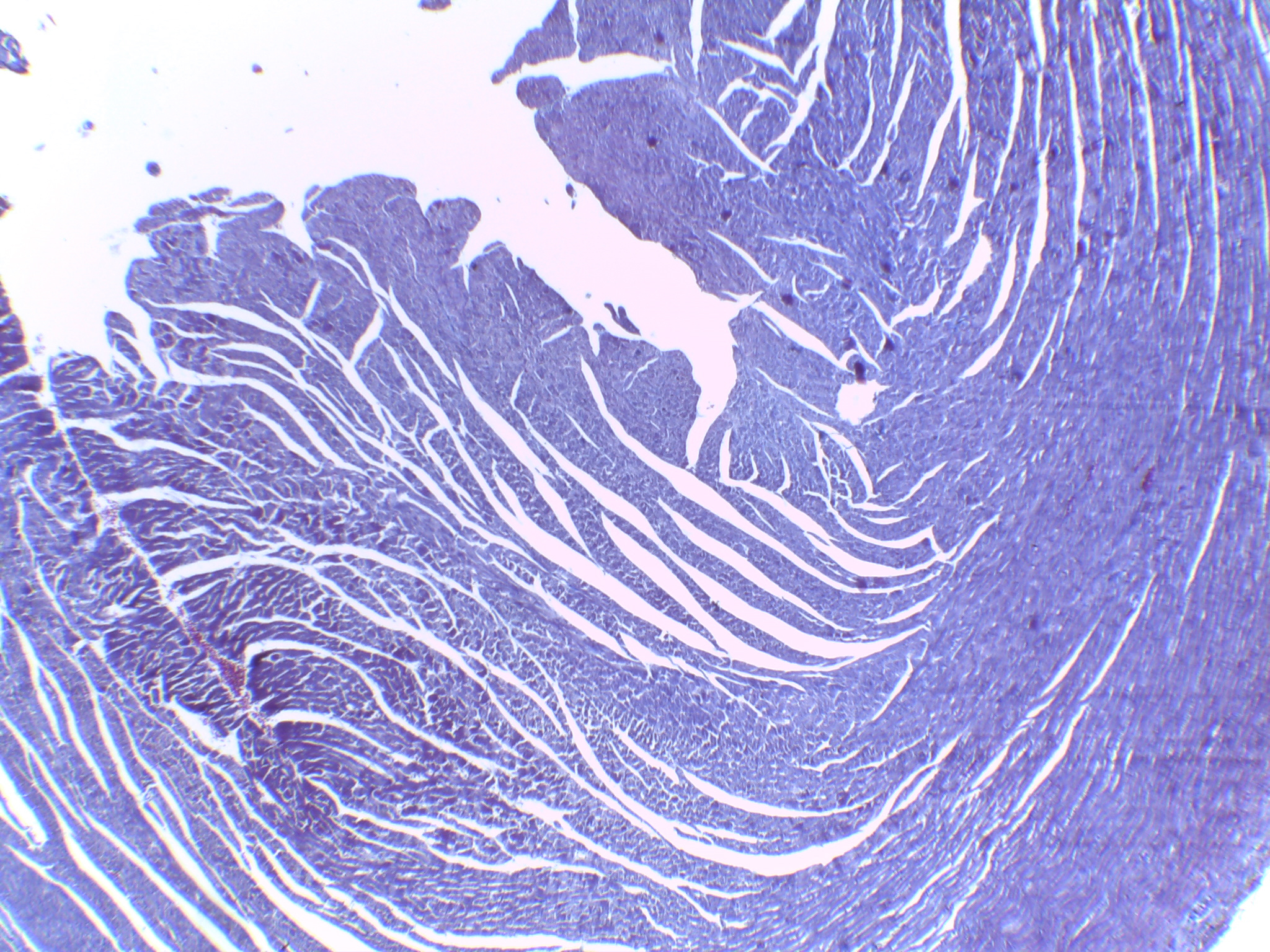

Supplement: Supplemental Information 6 [file peerj-12-17299-s006.zip › Raw data_Masson's /D gal/D-gal 4.jpg]

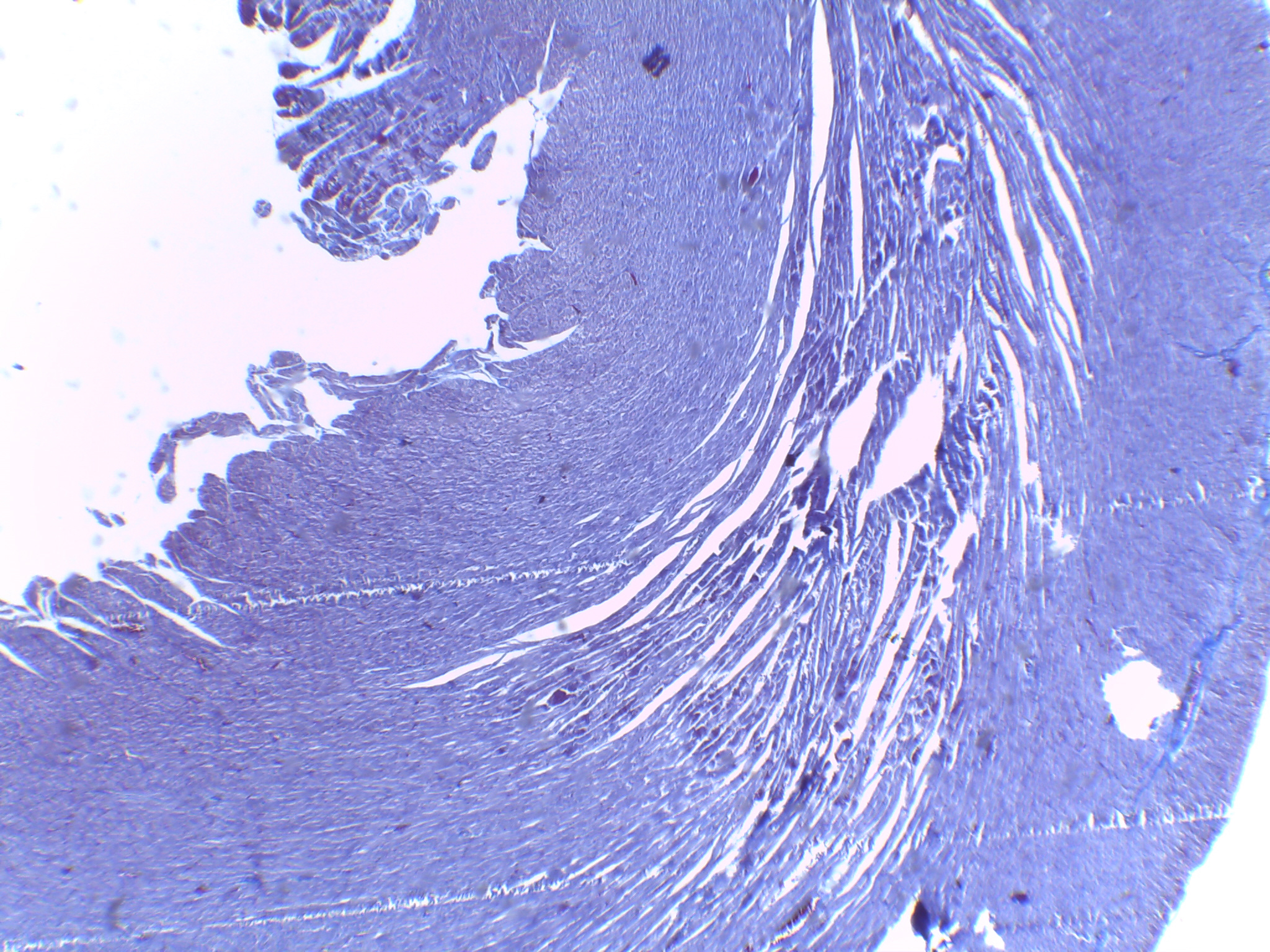

Supplement: Supplemental Information 6 [file peerj-12-17299-s006.zip › Raw data_Masson's /D gal/D-gal 2.jpg]

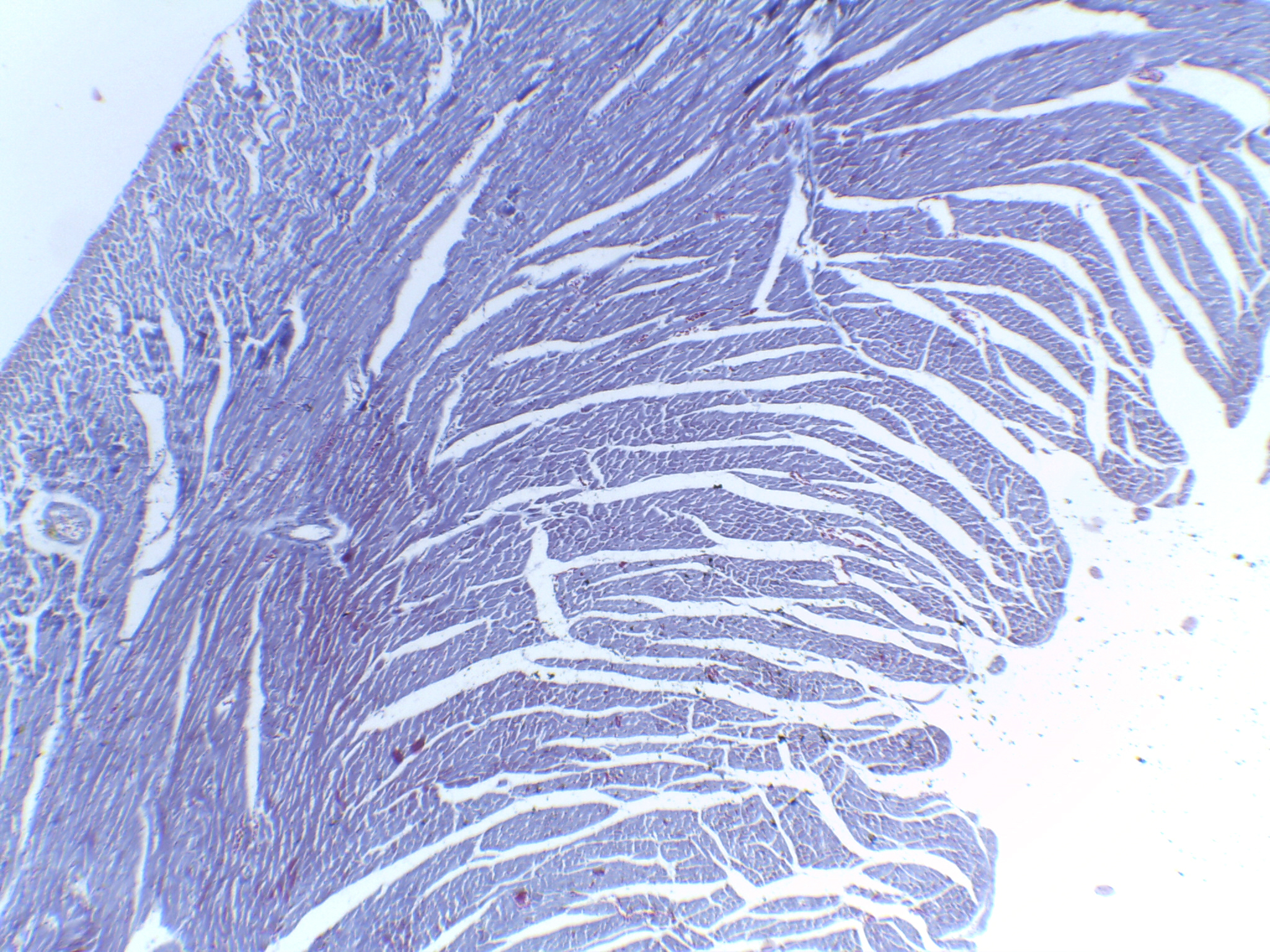

Supplement: Supplemental Information 6 [file peerj-12-17299-s006.zip › Raw data_Masson's /D gal/D-gal 3.jpg]

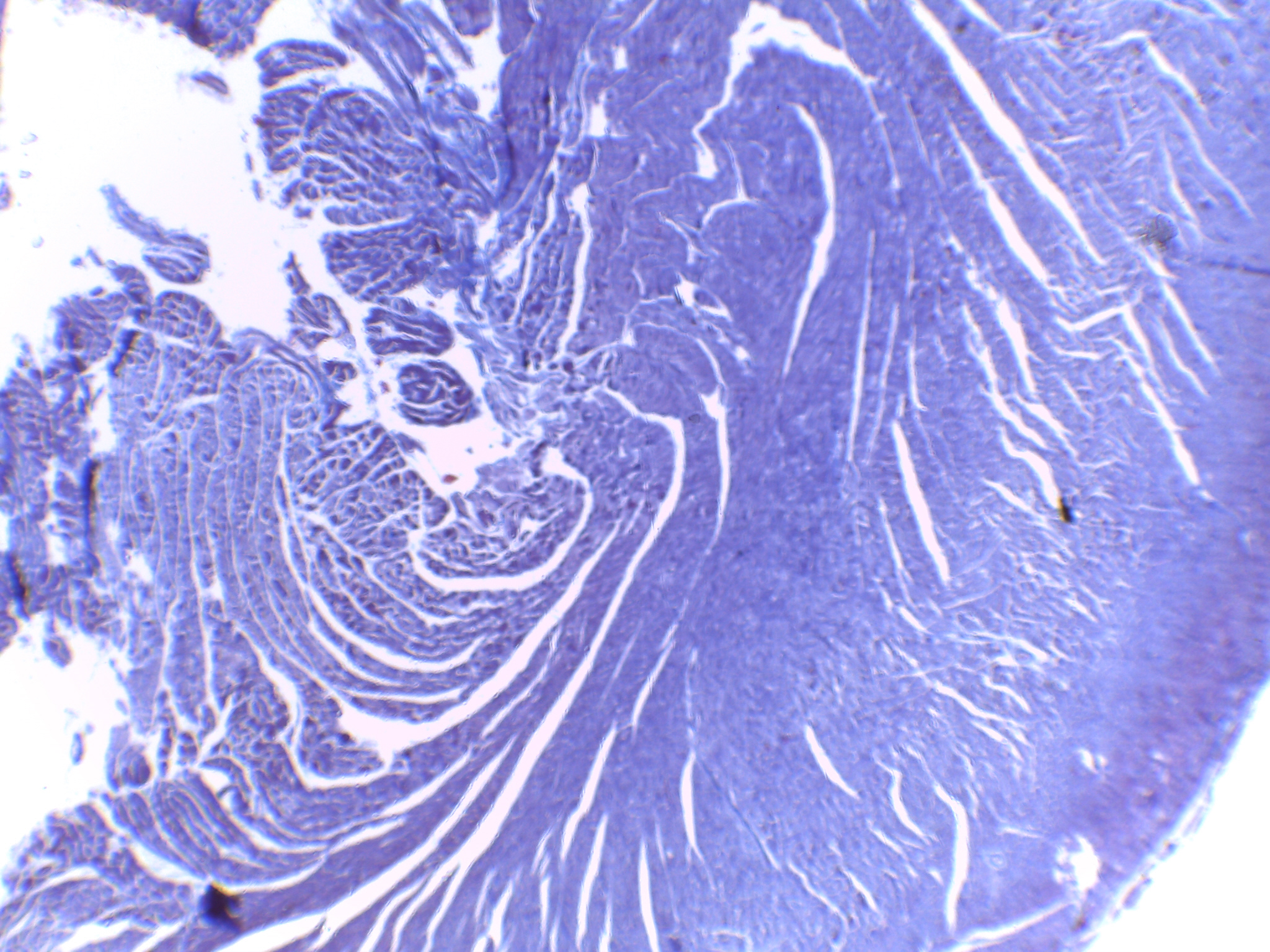

Supplement: Supplemental Information 6 [file peerj-12-17299-s006.zip › Raw data_Masson's /D gal/D-gal 1.jpg]

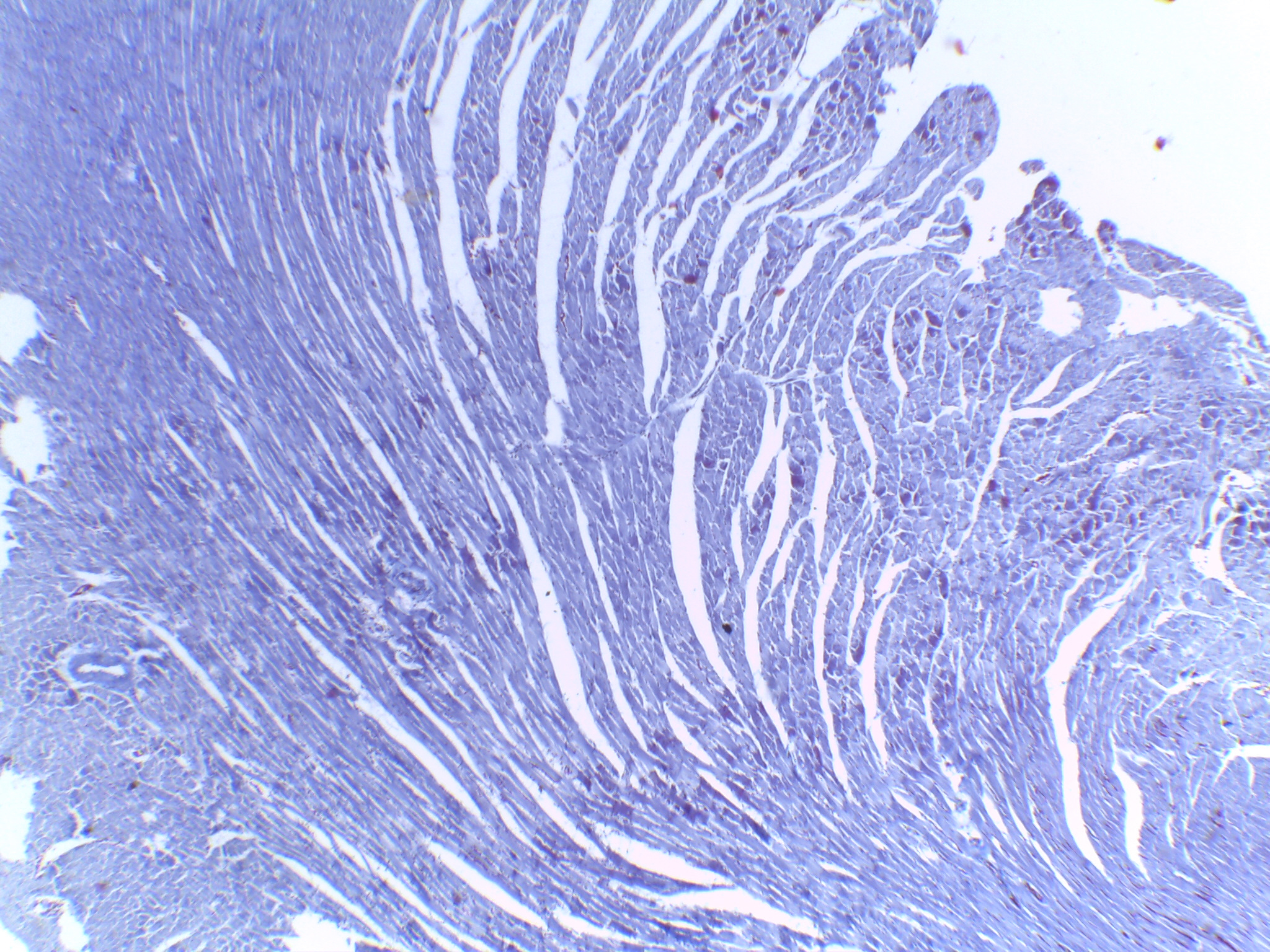

Supplement: Supplemental Information 6 [file peerj-12-17299-s006.zip › Raw data_Masson's /DPScs/D-gal+DPSCs 4.jpg]

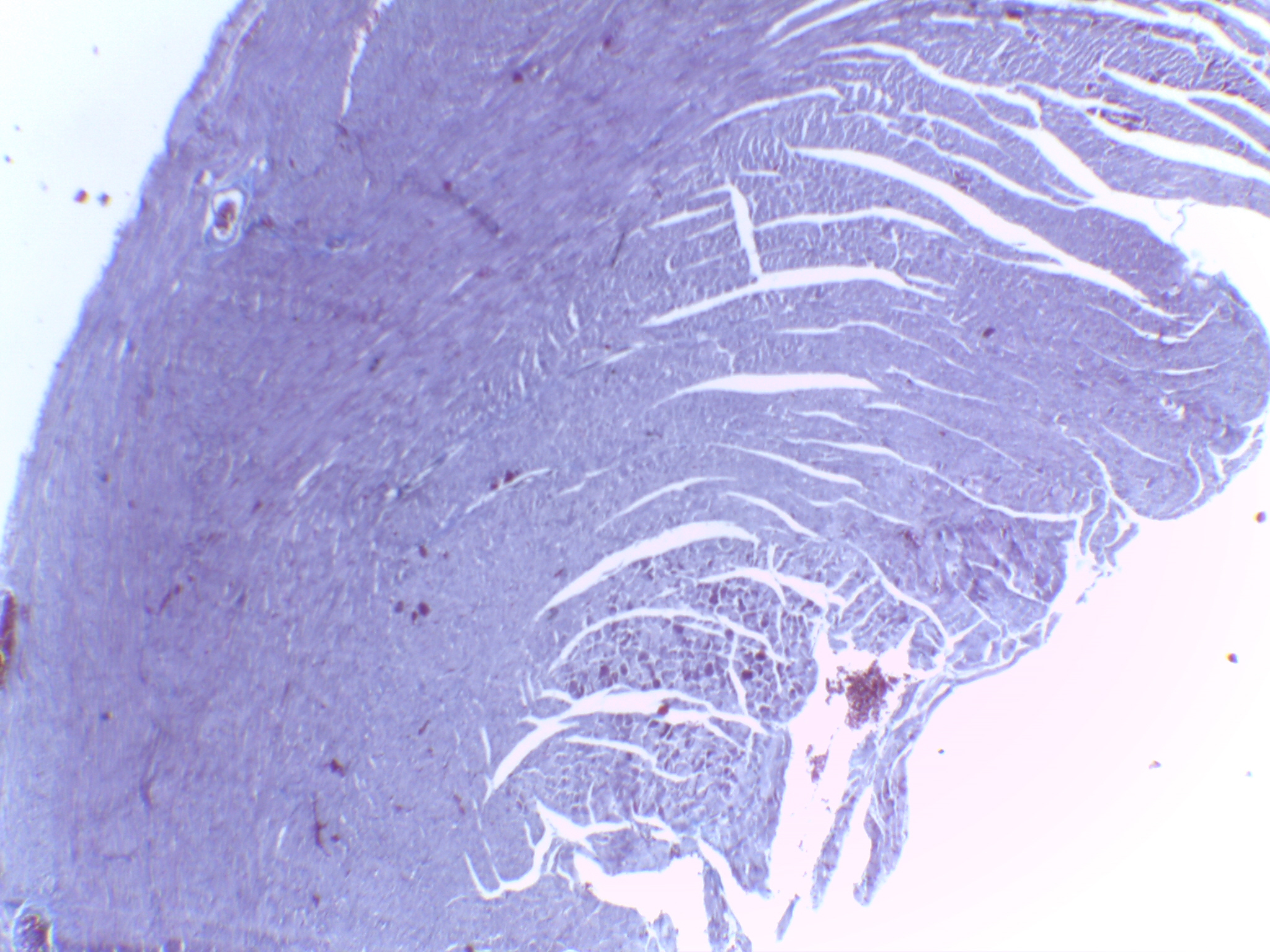

Supplement: Supplemental Information 6 [file peerj-12-17299-s006.zip › Raw data_Masson's /DPScs/D-gal+DPSCs 5.jpg]

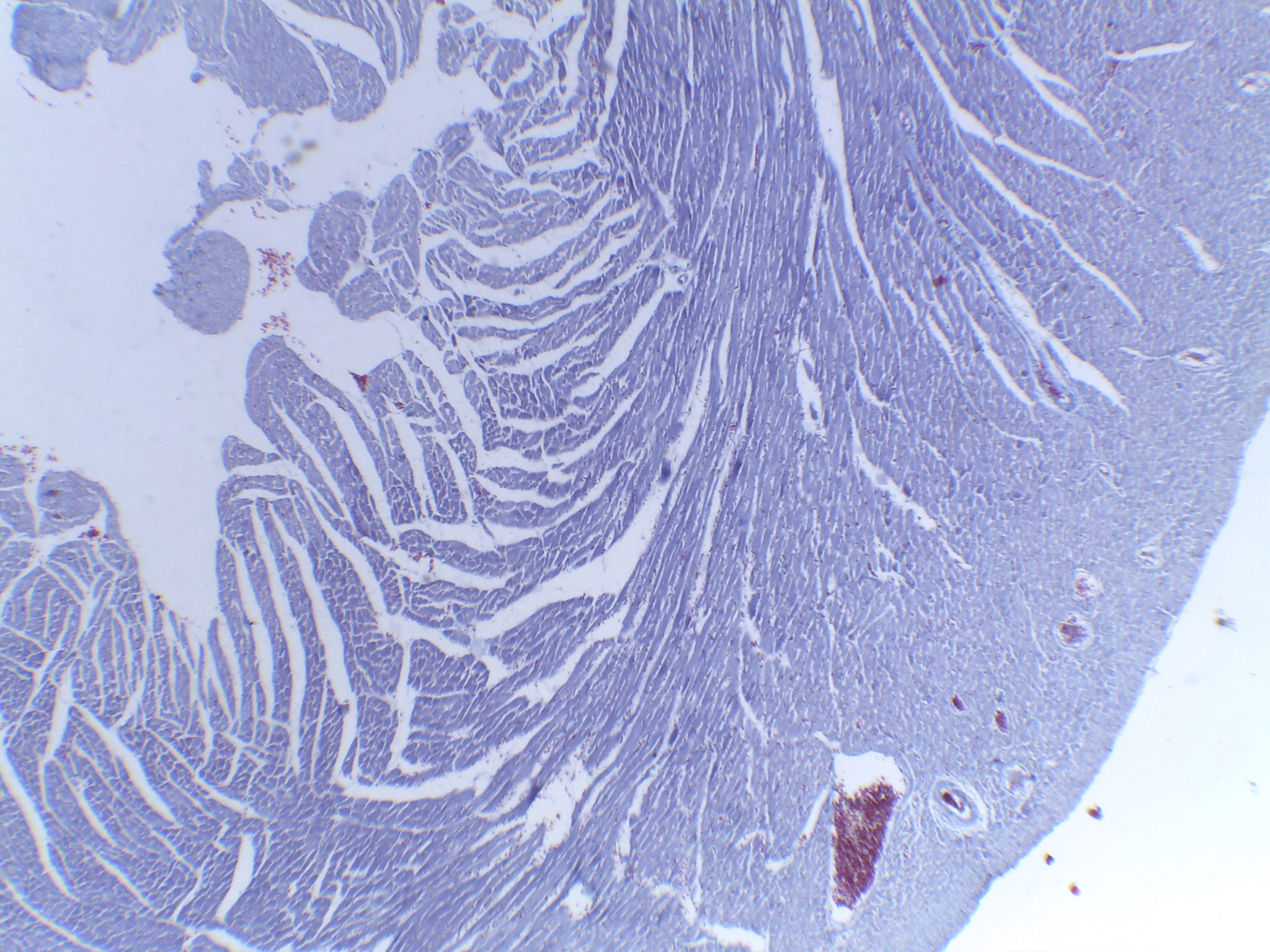

Supplement: Supplemental Information 6 [file peerj-12-17299-s006.zip › Raw data_Masson's /DPScs/D-gal+DPSCs 2.jpg]

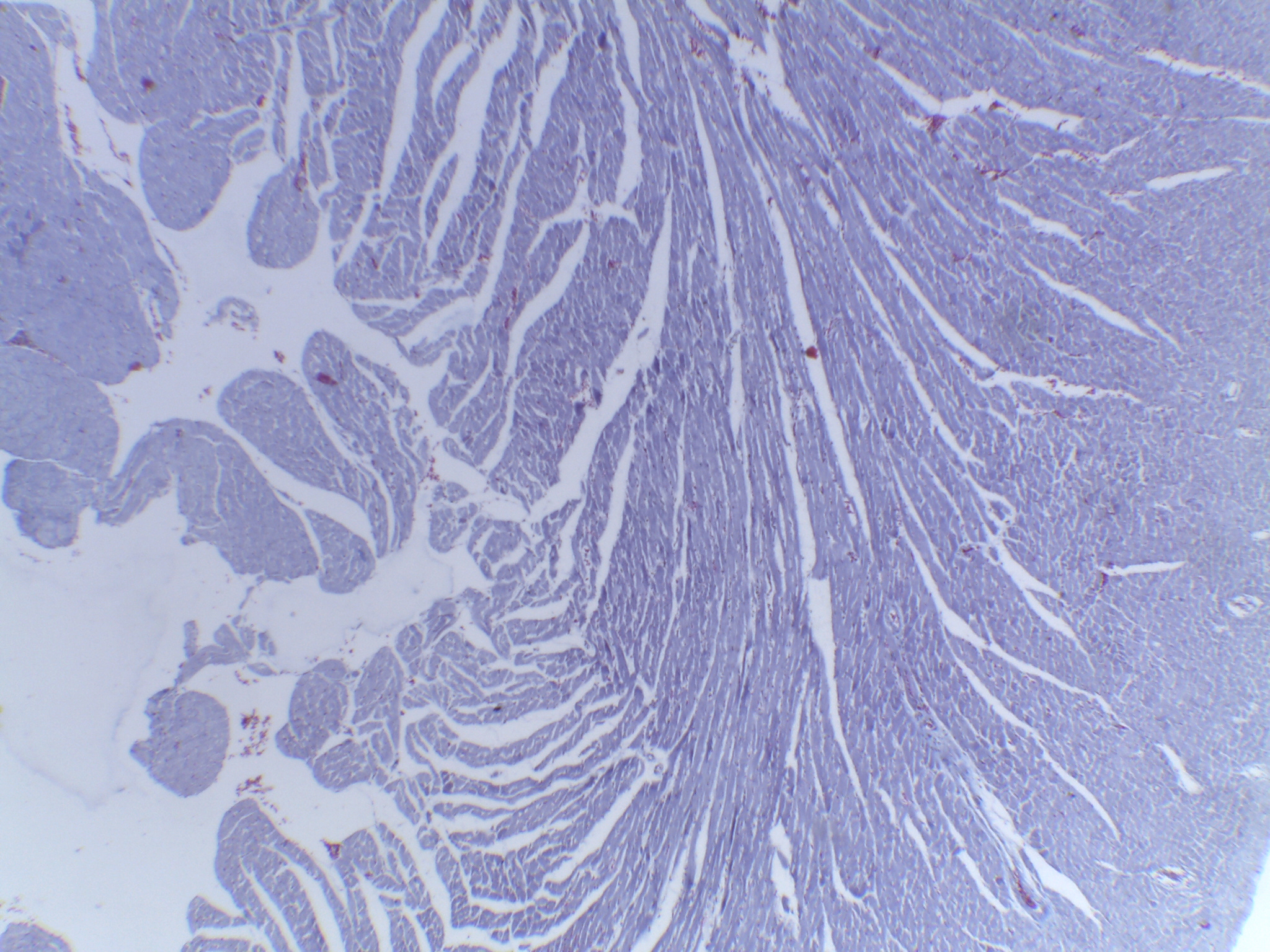

Supplement: Supplemental Information 6 [file peerj-12-17299-s006.zip › Raw data_Masson's /DPScs/D-gal+DPSCs 1.jpg]

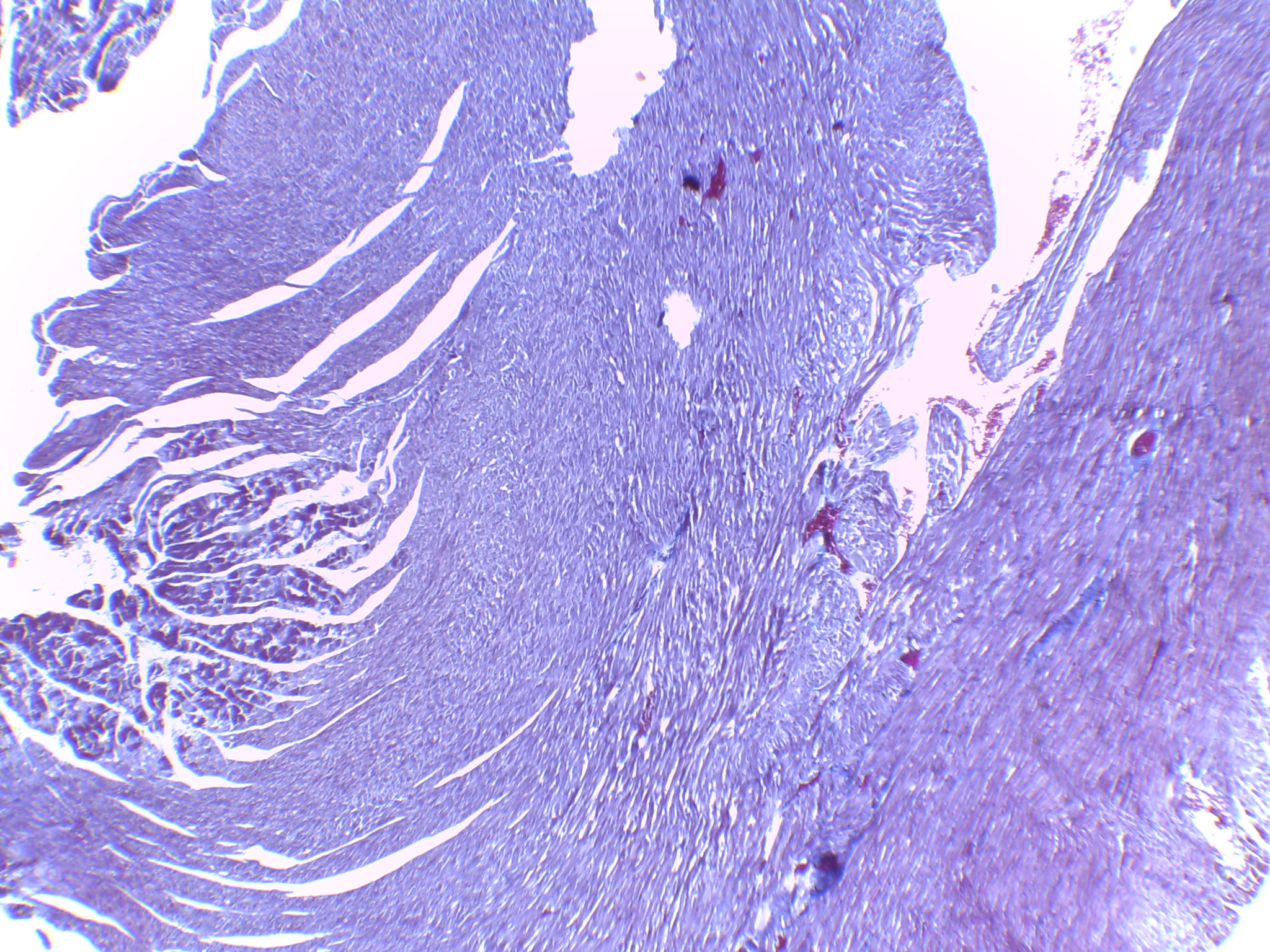

Supplement: Supplemental Information 6 [file peerj-12-17299-s006.zip › Raw data_Masson's /Control/Control 2.jpg]

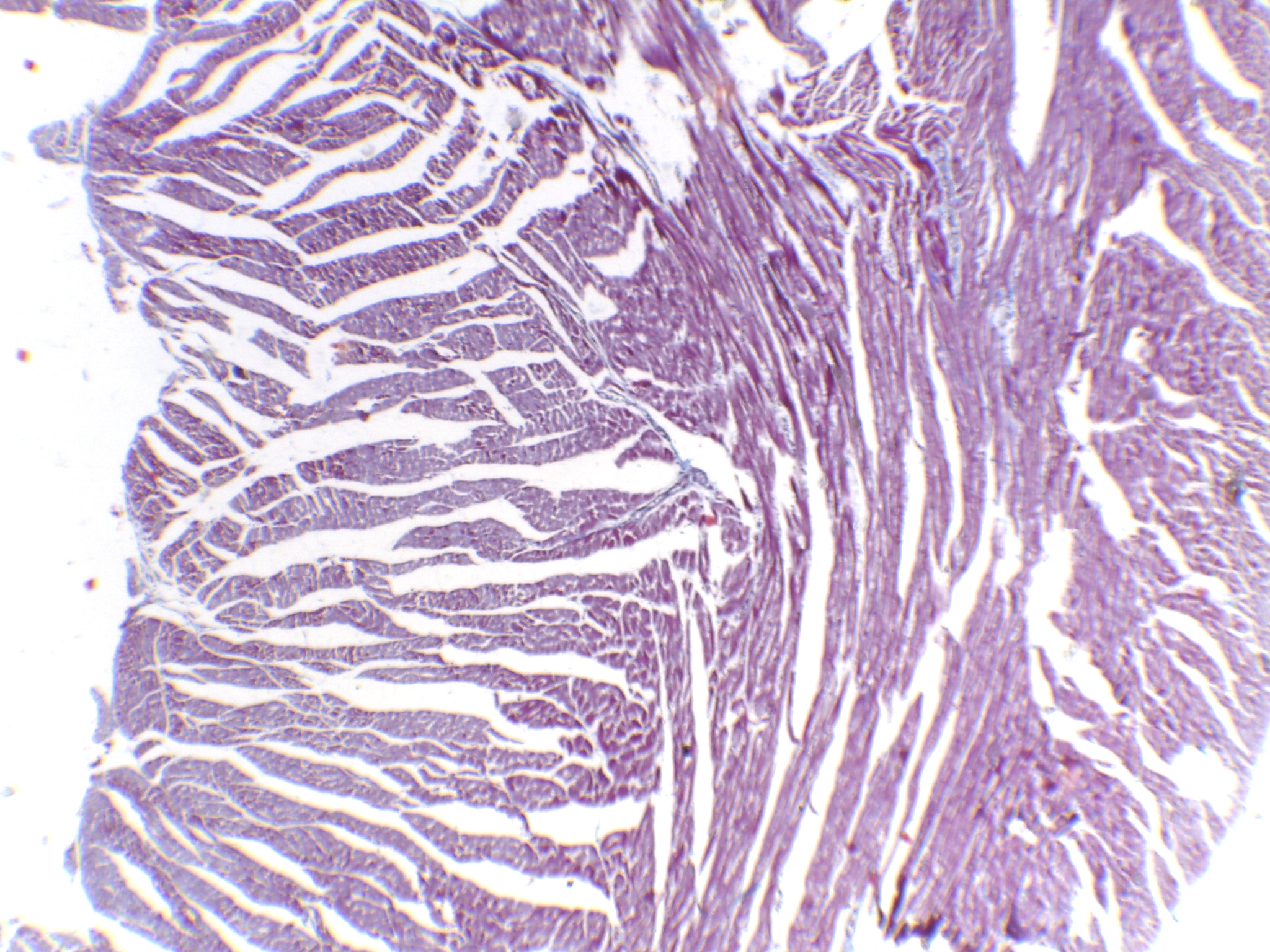

Supplement: Supplemental Information 6 [file peerj-12-17299-s006.zip › Raw data_Masson's /Control/Control 3.jpg]

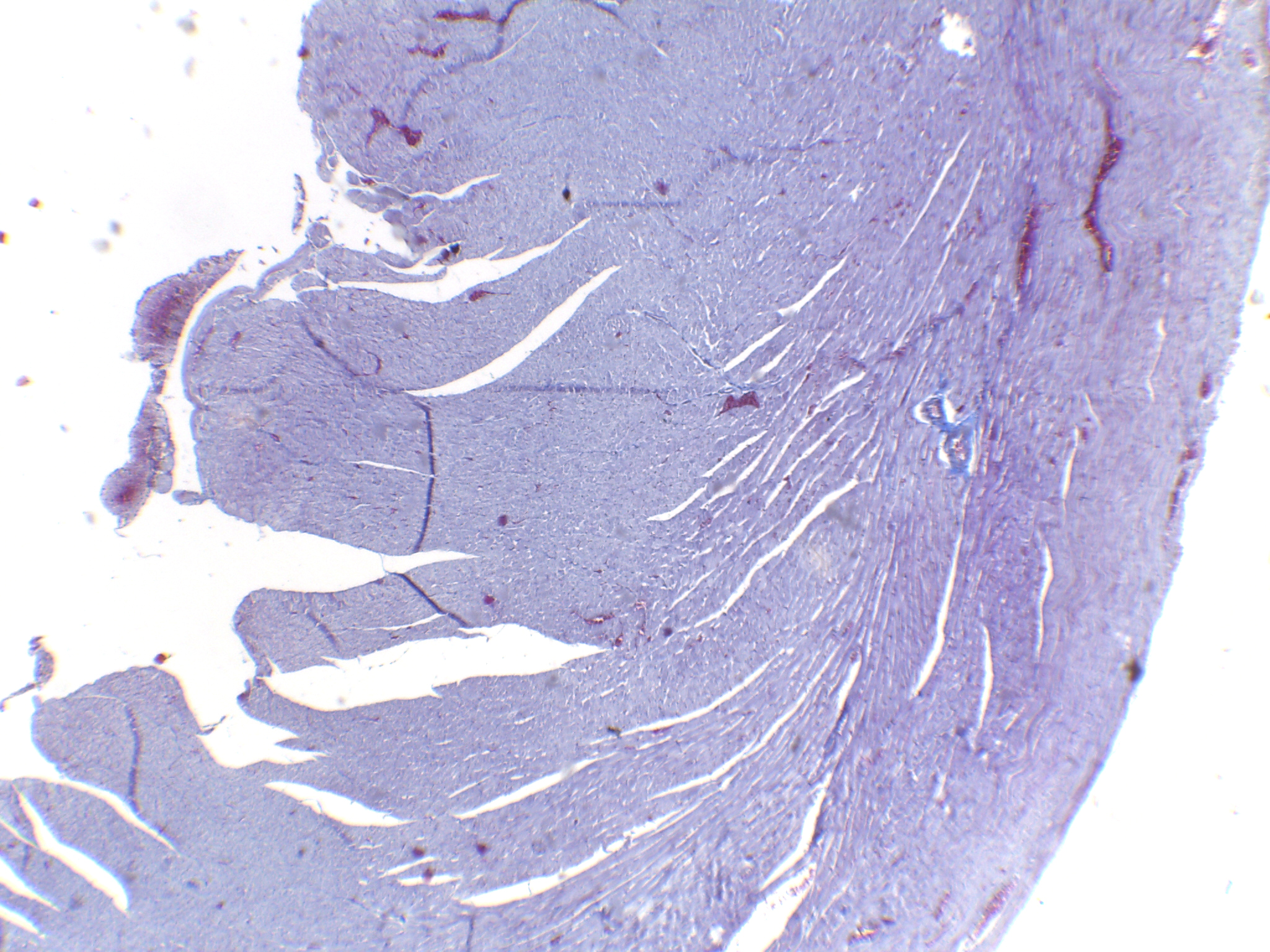

Supplement: Supplemental Information 6 [file peerj-12-17299-s006.zip › Raw data_Masson's /Control/Control 1.jpg]

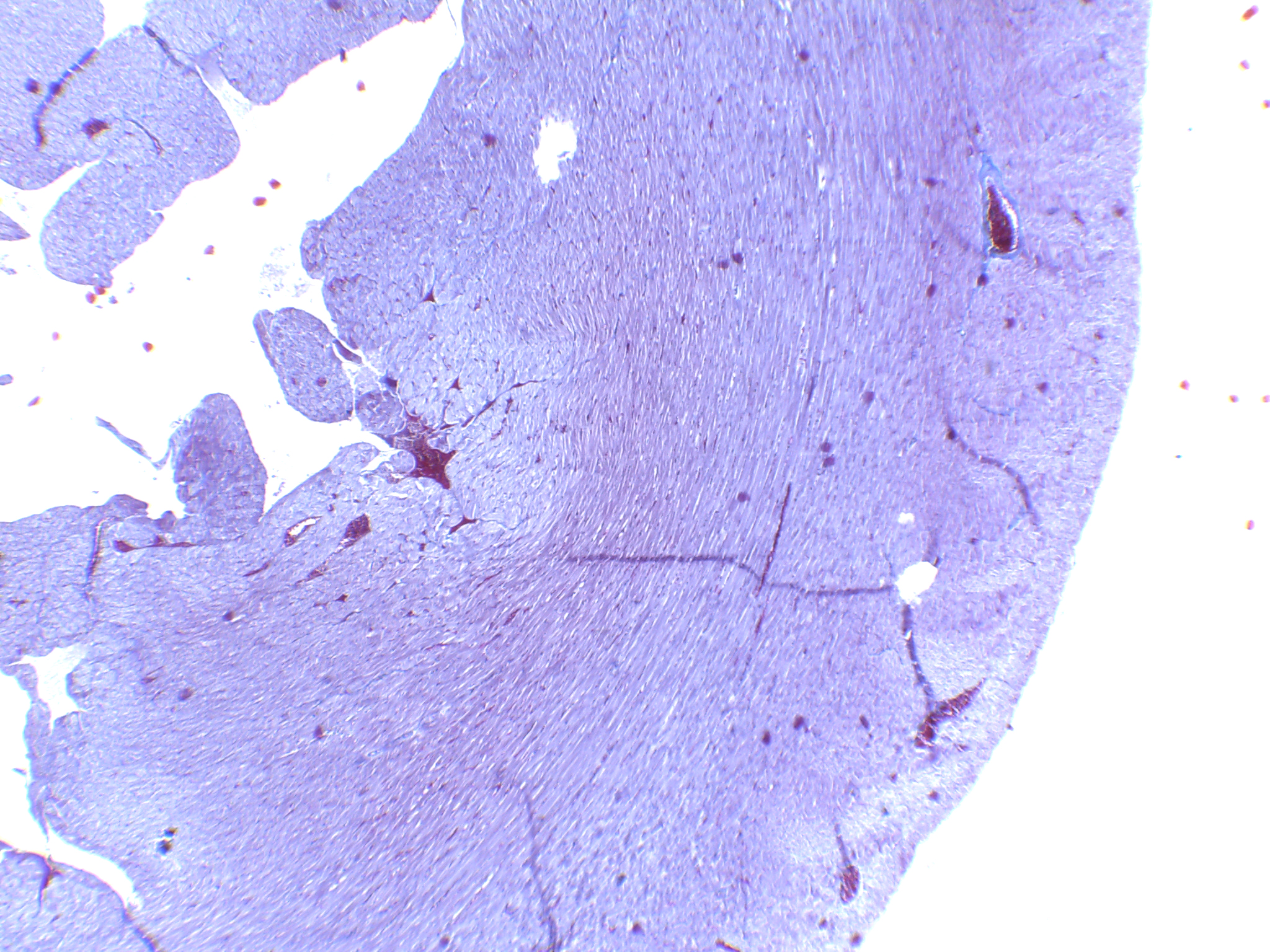

Supplement: Supplemental Information 6 [file peerj-12-17299-s006.zip › Raw data_Masson's /Control/Control 4.jpg]
