# Supplementary material for: Dental pulp stem cells ameliorate D-galactose-induced cardiac ageing in rats
Source: PeerJ. 2024 May 21;12:e17299. doi: 10.7717/peerj.17299 (PMC11127642; doi:10.7717/peerj.17299)
Supplement: Supplemental Information 7 [file peerj-12-17299-s007.pdf]

BAX

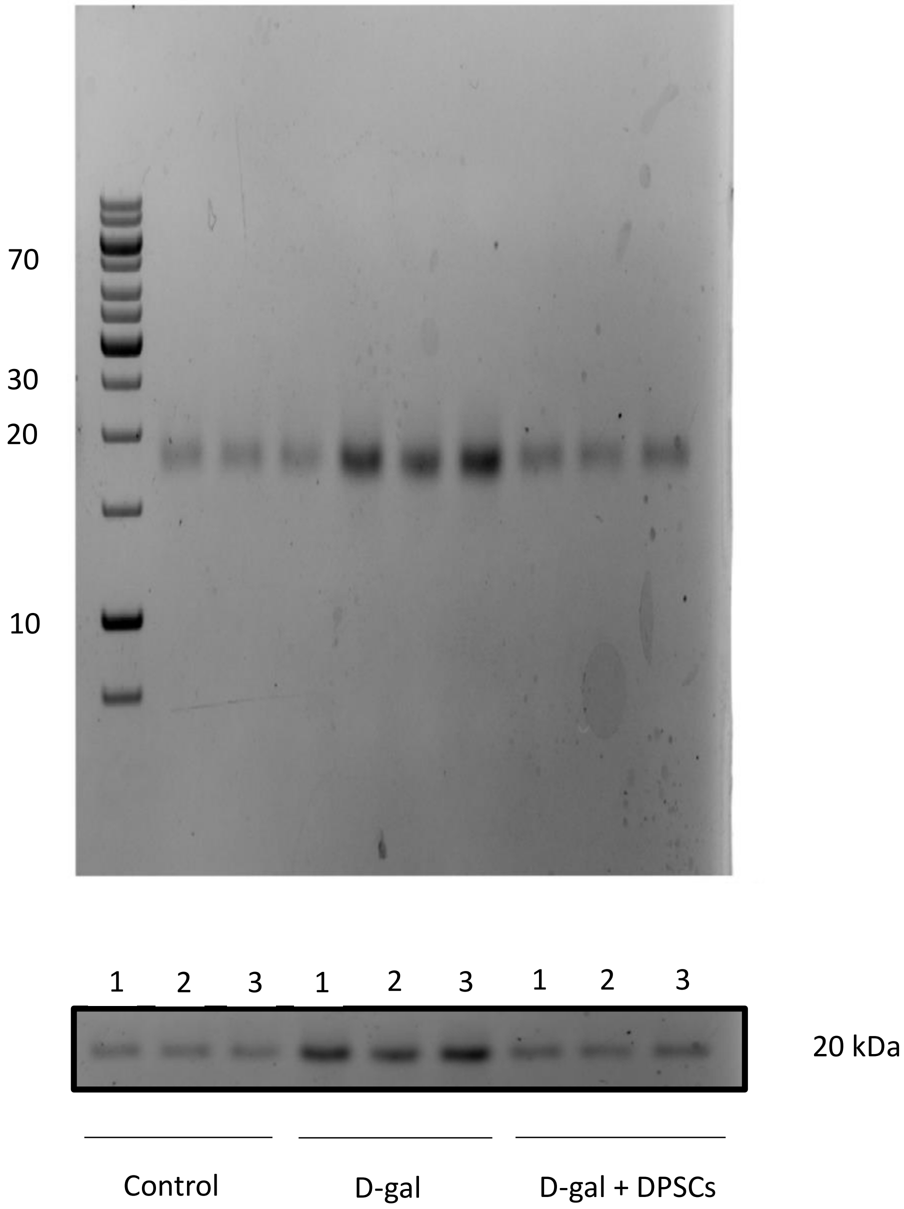

Each lane represents a biological replicate.

BCL2

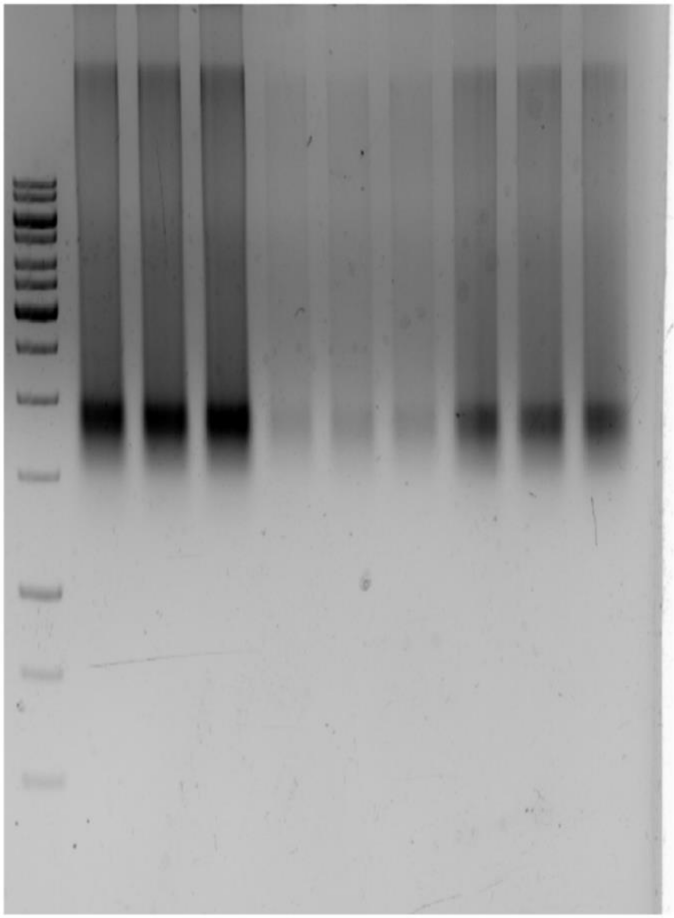

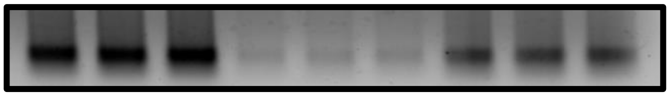

Each lane represents a biological replicate.

Caspase-3

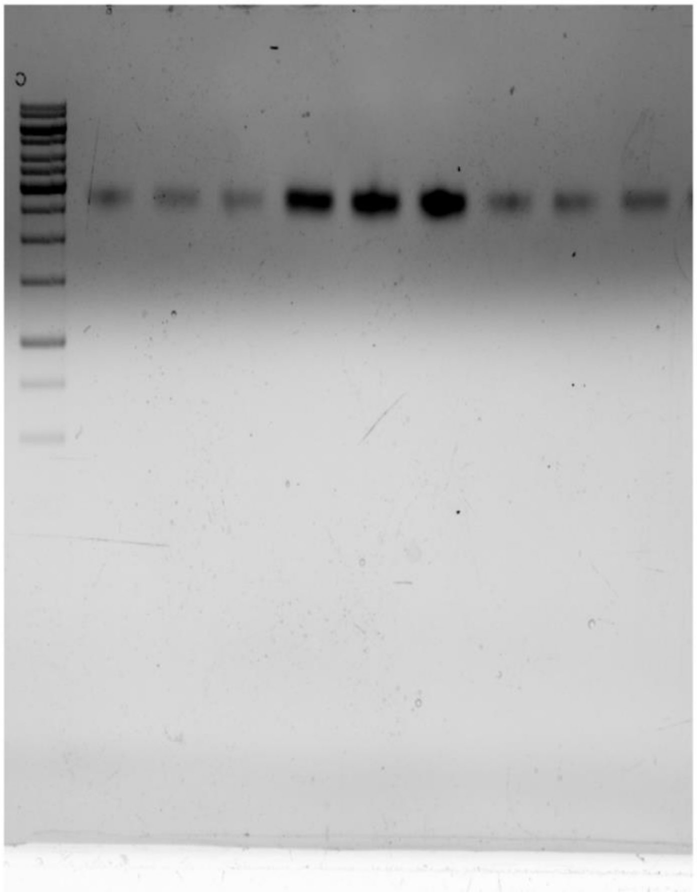

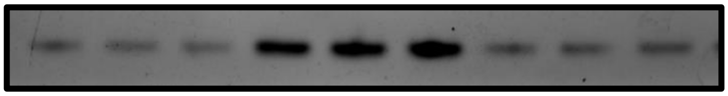

Each lane represents a biological replicate.

Cytochrome-c

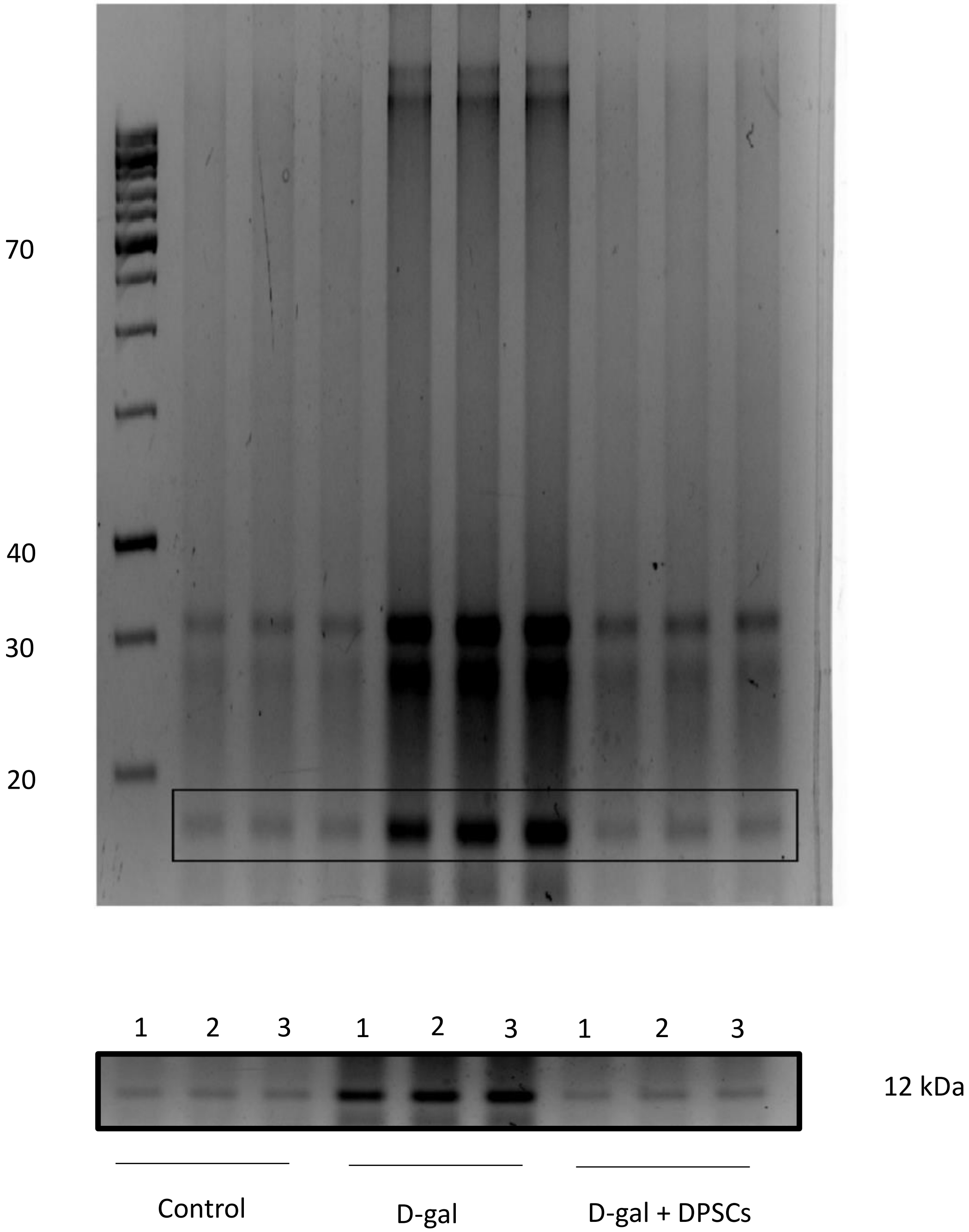

Each lane represents a biological replicate.

SIRT 1

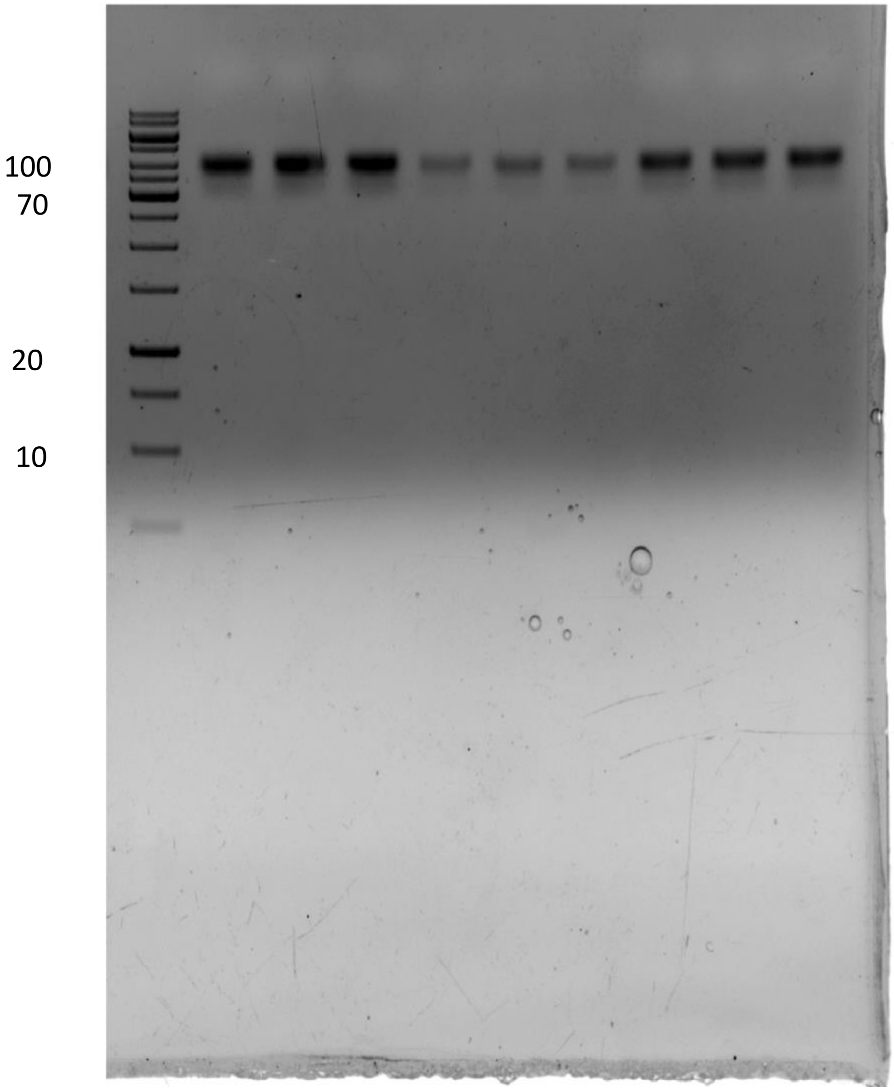

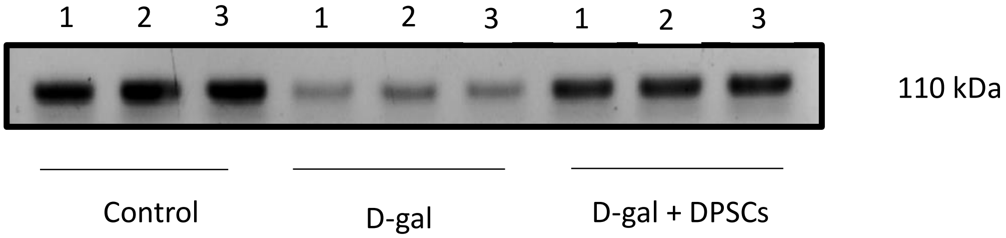

Each lane represents a biological replicate.

B Actin

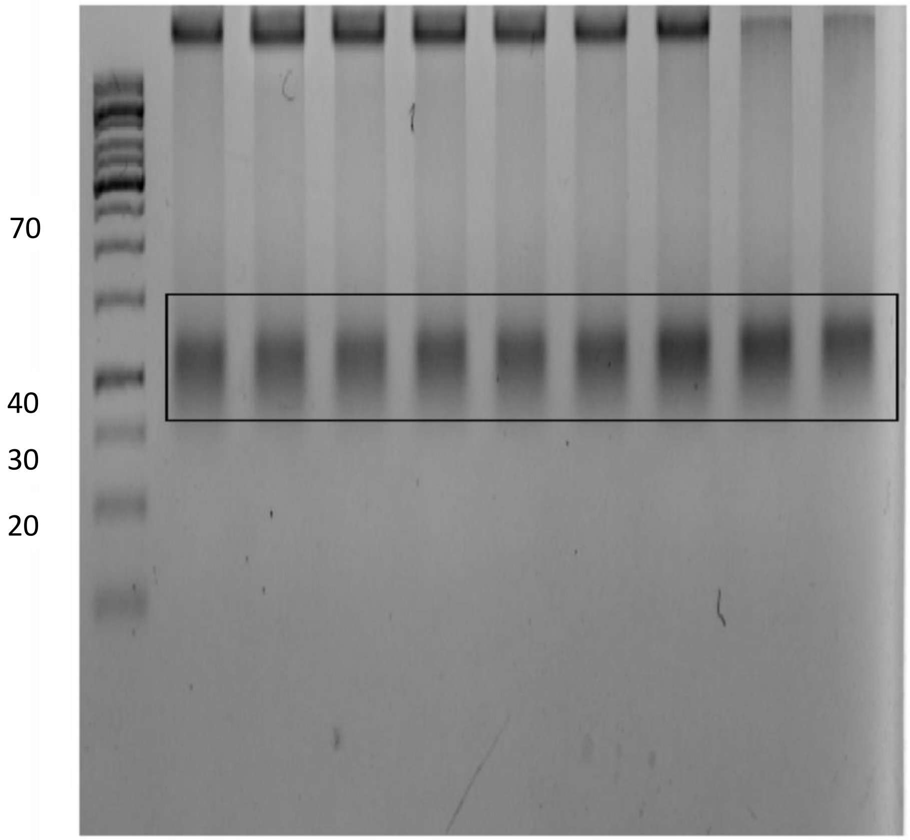

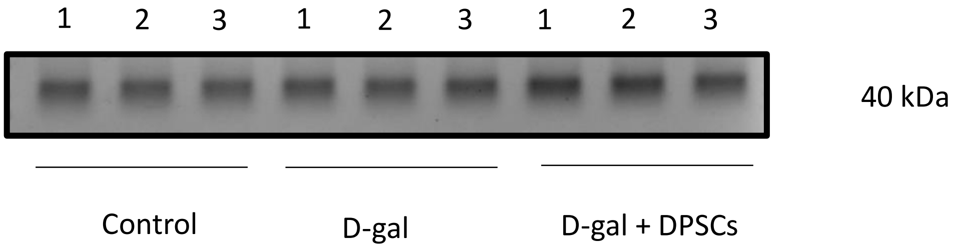

Each lane represents a biological replicate.
